# Syndecan-4 negatively regulates antiviral signalling by mediating RIG-I deubiquitination via CYLD

**DOI:** 10.1038/ncomms11848

**Published:** 2016-06-09

**Authors:** Wei Lin, Jing Zhang, Haiyan Lin, Zexing Li, Xiaofeng Sun, Di Xin, Meng Yang, Liwei Sun, Lin Li, Hongmei Wang, Dahua Chen, Qinmiao Sun

**Affiliations:** 1 State Key Laboratory of Membrane Biology, Institute of Zoology, Chinese Academy of Sciences, 1 Beichen West Road, Chaoyang District, Beijing 100101, China; 2 State Key Laboratory of Stem Cell and Reproductive Biology, Institute of Zoology, Chinese Academy of Sciences, 1 Beichen West Road, Chaoyang District, Beijing 100101, China

## Abstract

Retinoic acid-inducible gene I (RIG-I) plays important roles in pathogen recognition and antiviral signalling transduction. Here we show that syndecan-4 (SDC4) is a RIG-I-interacting partner identified in a yeast two-hybrid screen. We find that SDC4 negatively regulates the RIG-I-mediated antiviral signalling in a feedback-loop control manner. The genetic evidence obtained by using knockout mice further emphasizes this biological role of SDC4 in antiviral signalling. Mechanistically, we show that SDC4 interacts with both RIG-I and deubiquitinase CYLD via its carboxyl-terminal intracellular region. SDC4 likely promotes redistribution of RIG-I and CYLD in a perinuclear pattern post viral infection, and thus enhances the RIG-I–CYLD interaction and potentiates the K63-linked deubiquitination of RIG-I. Collectively, our findings uncover a mechanism by which SDC4 antagonizes the activation of RIG-I in a CYLD-mediated deubiquitination-dependent process, thereby balancing antiviral signalling to avoid deleterious effects on host cells.

## Abstract (web summary)

Syndecans are transmembrane proteoglycans implicated in diverse cellular activities. Here the authors show that Syndecan-4 via its cytosolic domain negatively regulates antiviral immunity by enhancing RIG-I interaction with a deubiquitinating enzyme CYLD, thus inhibiting the activating K63-linked RIG-I ubiquitination.

## Graphical abstract

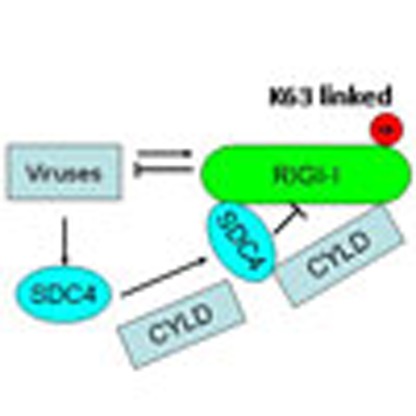

Innate immunity represents the first line of defence of host cells against invading pathogens, including viruses, bacteria and fungi. Detecting conserved microbial molecules, known as pathogen-associated molecular patterns (PAMPs), in host cells involves multiple distinct pattern recognition receptors that function in PAMP-specific and receptor-localized ways^1^. For example, membrane-bound Toll-like receptors recognize PAMPs in endosomes, whereas retinoic acid-inducible gene I (RIG-I)-like receptors (RLRs) and nucleotide-binding oligomerization domain-like receptors recognize PAMPs in cytosolic compartments on viral infection^2^,^3^.

The RIG-I receptor plays important roles in the cytosolic recognition of viral RNAs and in the regulation of the antiviral signalling pathway^4^,^5^. Structurally, RIG-I contains two amino-terminal caspase activation and recruitment domain (CARD) domains, and a carboxyl-terminal RNA helicase domain that is required for binding viral RNAs^3^. The recognition of viral RNA results in a conformational change in RIG-I that allows its CARD domains to be ubiquitinated by the E3 ligase TRIM25 via a K63 linkage, thereby leading to its activation^6^. Activated RIG-I physically interacts with the adaptor protein MAVS (also known as CARDIF, IPS-1 or VISA), which is located on the outer mitochondrial membrane, and consequently activates the downstream transcription factors IRF3 and NF-κB that induce the expression of type I interferons (IFNs) and pro-inflammatory cytokines^7^,^8^,^9^,^10^.

As is known, overproduction of pro-inflammatory cytokines potentially causes autoimmunity problems and diseases; thus, the regulation of inflammatory responses must be controlled to ensure that host cells maintain proper immune homeostasis. In terms of the regulation of RIG-I, previous studies have identified several ubiquitination-related factors that either positively or negatively regulate RIG-I activity^4^. In addition to the activation of RIG-I by TRIM25 (ref. 6) and Riplet/RNF135 (ref. 11) via K63-linked ubiquitination at its N-terminal region and C-terminal RD domain, respectively, K48-linked ubiquitination mediated by RNF125 has been shown to negatively regulate RIG-I by mediating its degradation^12^. Conversely, a number of deubiquitinating enzymes, including CYLD^13^, USP21 (ref. 14) and USP4 (ref. 15), are responsible for RIG-I deubiquitination, and thus control the RIG-I-mediated antiviral signalling. The K63-linked ubiquitination of RIG-I and its subsequent redistribution to the membrane in a perinuclear pattern have been proposed to be an important step in the process of antiviral signal transduction^16^; however, little is known about the molecular mechanism of this step and the role it plays in immune signal transduction.

Syndecans (SDCs) are transmembrane heparan sulfate proteoglycans that are normally present on the cell surface. SDCs have been reported to interact with extracellular matrix molecules and growth factors through their glycosaminoglycan chains^17^. Importantly, SDCs are essential for proper development and tissue homeostasis, as the mutation of certain genes encoding proteoglycans can cause severe developmental defects and is usually associated with diseases^17^,^18^. SDC family proteins have been reported to be involved in regulating a variety of cellular processes, such as cell adhesion^19^, migration^20^,^21^,^22^ and angiogenesis^23^. For example, SDC4 can interact with and activate protein kinase C, a key enzyme involved in signal transduction, suggesting that it plays an important role in modulating signalling pathways^24^,^25^. Previous studies have shown the roles of SDCs (for example, SDC-1/4) in controlling viral infections, suggesting a potential role of SDC family proteins in immune signalling^26^,^27^; however, the way in which SDCs are involved in the regulation of antiviral signalling remains largely unknown.

In this study, we identified SDC4 as a RIG-I-interacting factor in a yeast two-hybrid screen. We show that SDC4 expression is induced by viral infection, but it functions as a negative regulator that attenuates RIG-I activity, thereby maintaining antiviral signalling homeostasis in a feedback regulatory manner. We provide extensive biochemical evidence to demonstrate that SDC4, via its carboxyl-terminal intracellular domain, interacts with RIG-I and CYLD, thereby facilitating the interaction between RIG-I and CYLD. This interaction increases the K63-linked deubiquitination of RIG-I, thereby attenuating RIG-I-mediated signal transduction, and it contributes to maintaining the homeostasis of innate immune signalling.

## Results

### Identification of SDC4 as a RIG-I-interacting partner

**Figure 1:**
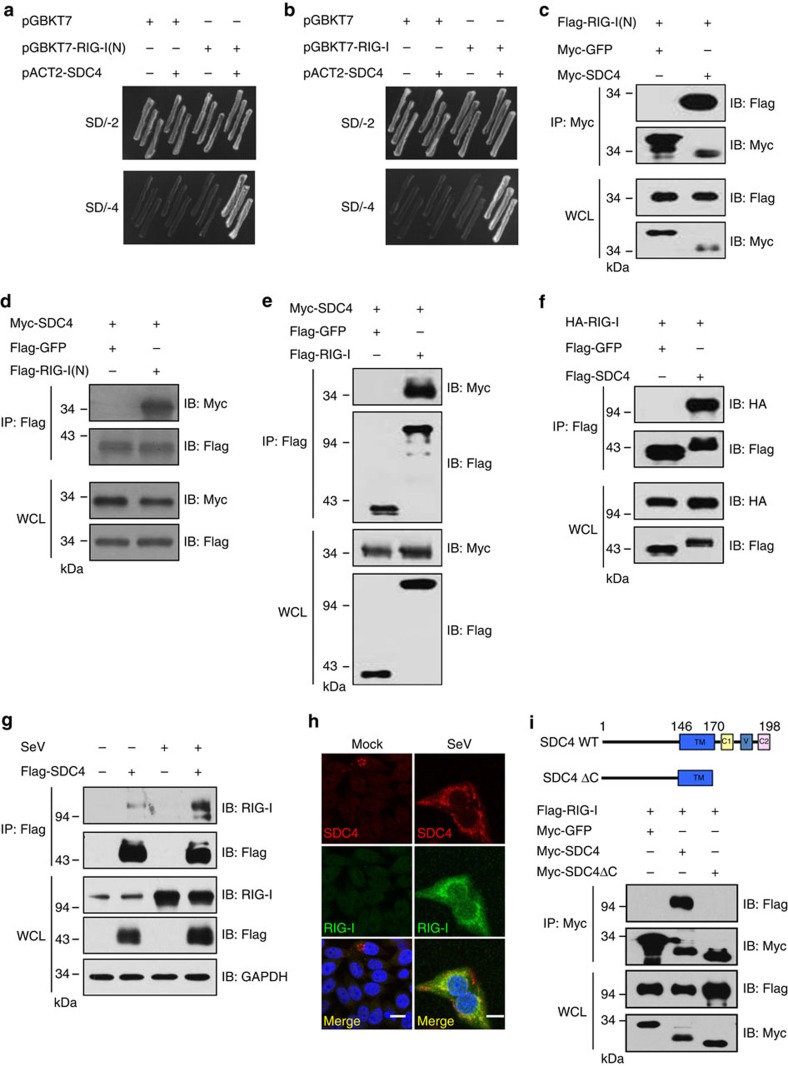
Identification of SDC4 as a protein that interacts with RIG-I. (**a**,**b**) The competent yeast strain AH109 was transformed with the indicated plasmids and plated on SD-Trp/-Leu plates and SD-Trp/-Leu/-His/-Ade plates. (**c**–**f**) HEK293 cells were transfected with the indicated expression plasmids. Twenty-four hours post transfection, the lysates were immunoprecipitated with anti-Myc beads (**c**) or anti-Flag beads (**d**–**f**), and analysed by immunoblotting with the indicated antibodies. Bottom panel, expression of exogenous proteins in whole-cell lysates (WCL). (**g**) HEK293 cells were transfected with an empty vector or a Flag-tagged SDC4 vector. Twenty-four hours post transfection, the cells were infected with SeV for 7 h, lysed, and immunoprecipitated with anti-Flag beads. Immunoprecipitates were analysed by immunoblotting with the indicated antibodies. (**h**) HeLa cells were left untreated or infected with SeV for 14 h, then fixed, stained with DAPI, anti-SDC4 and anti-RIG-I antibodies, and observed by confocal microscopy. Scale bar, 10 μM. (**i**) Structure diagram of SDC4 (upper panel). SDC4 interacts with RIG-I through its CP domain (lower panel). HEK293 cells were transfected with the indicated expression plasmids. Twenty-four hours post transfection, the lysates were immunoprecipitated with anti-Myc beads, and analysed by immunoblotting with the indicated antibodies. The data shown in **a**–**i** are from one representative experiment of at least three independent experiments.

To understand the molecular basis of how RIG-I-mediated innate immune signalling is regulated, we performed a yeast two-hybrid screen to search for RIG-I-binding partners using the RIG-I CARD domains as bait. From this screening, we found that one of the positive clones encodes human SDC4 (Fig. 1a). Consistently, we found SDC4 also interacted with the full-length form of RIG-I in yeast two-hybrid assays, as yeast carrying vectors expressing these two proteins could grow on a high selection pressure medium (Fig. 1b). To further confirm this interaction, we conducted co-immunoprecipitation (co-IP) experiments in human embryonic kidney 293 (HEK293) cells that expressed epitope-tagged SDC4 and the amino-terminal domain of RIG-I (RIG-I(N)) or full-length of RIG-I. As shown in Fig. 1c–f, SDC4 and RIG-I(N) or full-length RIG-I were reciprocally co-immunoprecipitated in transfected HEK293 cells. Since the SDC family proteins contain several members, namely, SDC1, SDC2, SDC3 and SDC4, to investigate the specificity of the interaction between RIG-I and SDC4, we tested whether RIG-I also interacts with SDC1, SDC2 or SDC3 by performing co-IP experiments. As shown in Supplementary Fig. 1a,b, in contrast to SDC4, SDC1, SDC2 and SDC3 failed to interact with RIG-I, indicating the specific interaction between SDC4 and RIG-I. Next, we determined whether the endogenous RIG-I protein could form a complex with SDC4. As shown in Fig. 1g and Supplementary Fig. 2, while endogenous RIG-I was only weakly present in both endogenous and overexpressed SDC4 complex in cultured cells under a normal condition, a relatively stronger SDC4–RIG-I interaction was detected on Sendai virus (SeV) infection. In line with this observation, RIG-I levels were significantly increased with SeV infection. In addition, our immunostaining assays showed that significant overlapping signals between endogenous SDC4 and RIG-I could be detected after HeLa cells were infected with Sendai virus (Fig. 1h). Given that SDC4 mainly contains a transmembrane (TM) domain and a cytoplasmic (CP) domain (Fig. 1i) and that SDC4 normally interacts with its interacting partners through its CP domain^20^,^28^,^29^, we sought to determine whether SDC4 interacts with RIG-I through its CP domain. As shown by co-IP assays, the CP domain is essential for the SDC4–RIG-I interaction, as the deletion of the CP domain of SDC4 (SDC4ΔC) completely abolished the SDC4–RIG-I interaction (Fig. 1i), whereas the mutant form of SDC4 (SDC4ΔTM), in which the transmembrane domain was deleted, still interacted with RIG-I (Supplementary Fig. 3a).

### SDC4 overexpression suppresses the RIG-I antiviral signalling

**Figure 2:**
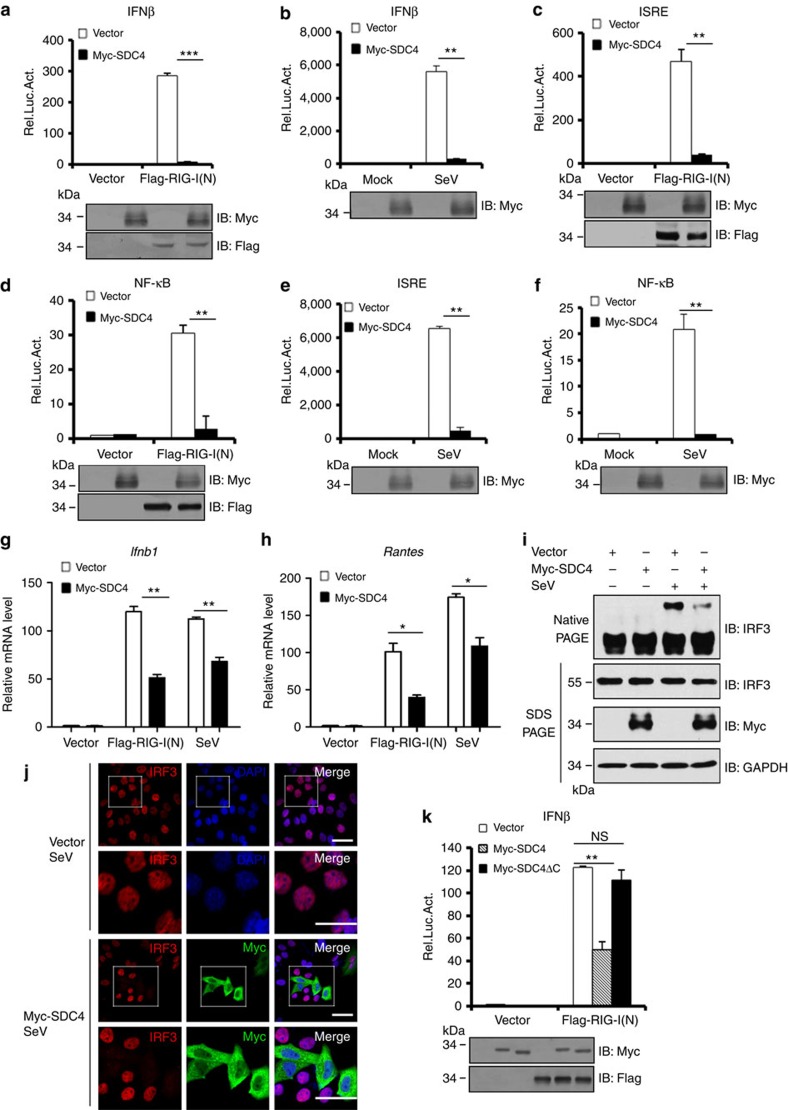
Overexpression of SDC4 inhibits RIG-I-mediated antiviral signaling. (**a**,**c**,**d**) HEK293 cells were co-transfected with the indicated expression plasmids, luciferase reporter constructs driven by the promoter of gene encoding IFN-β (**a**), ISRE (**c**) or NF-κB (**d**) and pRSV/LacZ as an internal control. Twenty-four hours after transfection, the cells were lysed for luciferase assays (upper panel) and immunoblotting assays (lower panels). (**b**,**e**,**f**) HEK293 cells were transfected with luciferase reporter constructs driven by the promoter of gene encoding IFN-β (**b**), ISRE (**e**) or NF-κB (**f**), and pRSV/LacZ as an internal control. Twenty-four hours after transfection, the cells were infected with SeV for 16 h, and then lysed for luciferase assays (upper panel) and immunoblotting assays (lower panel). (**g**,**h**) HEK293 cells were transfected with the indicated expression plasmids. Eighteen hours post transfection, the cells were left untreated or infected with SeV for 6 h, and the cell lysates were analysed by quantitative real-time PCR to examine the levels of the *Ifnb1* (**g**) or *Rantes* (**h**) transcripts. (**i**) HEK293 cells were transfected with the indicated expression plasmids. Twenty-four hours after transfection, the cells were left untreated or infected with SeV for 9 h. The lysates were resolved by native gel electrophoresis (upper panel) or SDS–PAGE (lower panels) and analysed with the indicated antibodies. (**j**) HeLa cells were transfected with the indicated expression plasmids. Twenty-four hours after transfection, the cells were infected with SeV for 9 h, stained with the indicated antibodies and DAPI, and then imaged by confocal microscopy. Bottom panels were the larger magnification. Scale bar, 50 μM. (**k**) HEK293 cells were co-transfected with the indicated expression plasmids. Twenty-four hours after transfection, the cells were lysed for luciferase assays (upper panel) and immunoblotting assays (lower panels). The data shown in **a**–**h** and **k** are from one representative experiment of at least three independent experiments (mean±s.d. of duplicate experiments in **a**–**f** and **k** or triplicate experiments in **g** and **h**). The two-tailed Student's *t*-test was used to analyse statistical significance. *P<0.05; **P<0.01; ***P<0.001; NS, not significant versus control groups.

Having determined that SDC4 forms a complex with RIG-I in cells, we sought to examine whether SDC4 has a role in regulating RIG-I-mediated immune signalling. First, we tested whether SDC4 overexpression affects the activation of the type I IFN promoter that is induced by RIG-I(N), a constitutively active form of RIG-I. As shown in Fig. 2a, RIG-I(N) expression was sufficient to trigger the activation of the IFN-β promoter in transfected HEK293 cells. In contrast, co-expressing SDC4 and RIG-I(N) in HEK293 cells significantly reduced RIG-I(N)-mediated IFN-β luciferase activity. Consistently, we found that increased SDC4 expression also suppressed IFN-β luciferase expression in response to SeV infection (Fig. 2b). Because IFN-β activation requires coordinated signalling from both the IRF3- and NF-κB-mediated pathways^30^, we employed an IFN-stimulated response element (ISRE) luciferase reporter, which could be sufficiently activated by IRF3 activation, and an NF-κB luciferase reporter, and examined how SDC4 attenuates IFN-β signal transduction. As shown in Fig. 2c–f and Supplementary Fig. 4a-b, SDC4 overexpression significantly suppressed the activation of the ISRE and NF-κB promoters following RIG-I(N) expression or SeV infection; however, it had no effects on the activation of the IFN-β or ISRE promoter following MDA5(N) (an active form of MDA5) expression, suggesting that SDC4 specifically targets RIG-I to attenuate its antiviral signalling activity. Similarly, we also found that SDC4 expression significantly reduced the levels of transcripts of antiviral genes, such as *Ifnb1* and *Rantes*, following RIG-I(N) expression or SeV infection (Fig. 2g,h). To further understand how SDC4 inhibits the type I IFN-β pathway, we assessed whether SDC4 expression affects the dimerization and nuclear translocation of IRF3, which are important features of the activation of RLR pathways, when HEK293 cells were infected with SeV. As shown in Fig. 2i,j, SDC4 overexpression markedly inhibited the dimerization and nuclear translocation of IRF3 following SeV infection. Following RIG-I(N) overexpression, SDC4 overexpression consistently inhibited IRF3 dimerization induced by RIG-I(N) expression in a dose-dependent manner, which further confirmed that SDC4 plays an important role in inhibiting RIG-I-mediated activation (Supplementary Fig. 5). Because SDC4 interacts with RIG-I through its CP domain, we tested whether SDC4ΔC affects the RIG-I(N)-mediated activation of the IFN-β promoter. As shown in Fig. 2k, SDC4ΔC expression failed to inhibit the RIG-I(N)-induced activation of the IFN-β promoter, suggesting that the carboxyl terminus of SDC4 is required to inhibit the activation of the IFN-β promoter. Taken together, these findings suggest that SDC4 potentially antagonizes RIG-I-mediated antiviral signalling through its CP domain. In addition, we found that deletion of TM damaged the function of SDC4 in inhibiting the RIG-I-mediated signalling (Supplementary Fig. 3b), suggesting that the TM domain is important for SDC4 to regulate the RIG-I signalling.

### SDC4 knockdown enhances the RIG-I antiviral signalling

**Figure 3:**
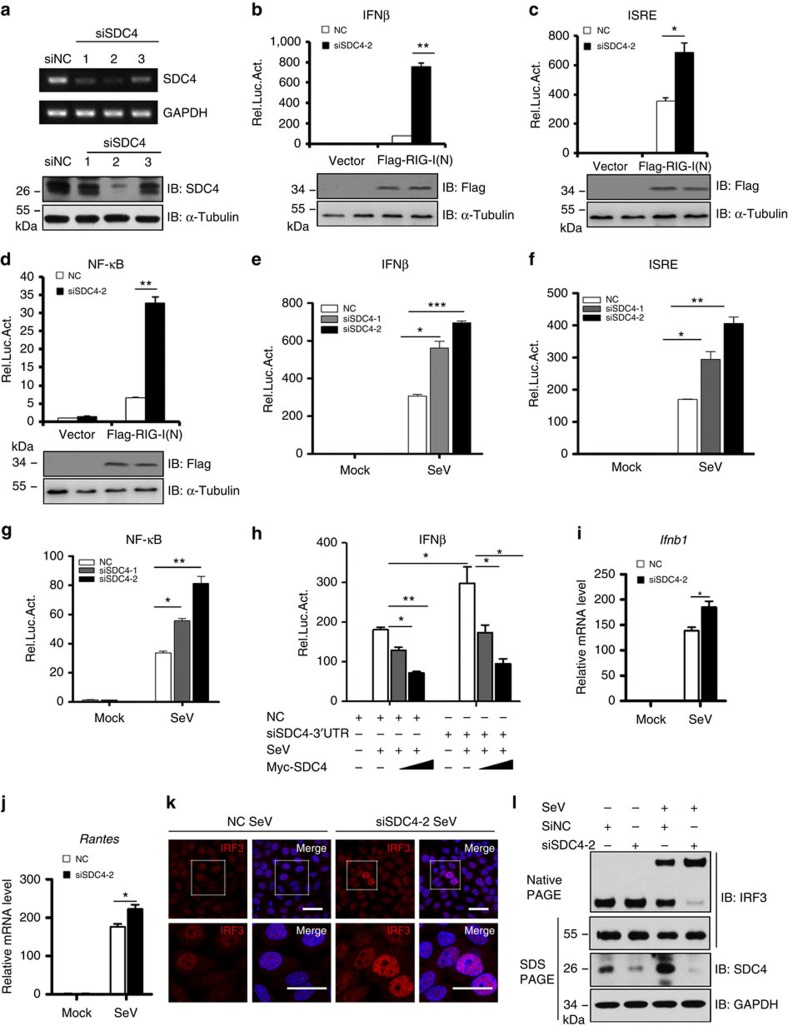
***Sdc4*** **knockdown potentiates RIG-I signalling.** (**a**) HEK293 cells were transfected with siRNA targeting different *Sdc4* regions or a non-targeting control (NC). Forty-eight hours later, the cell lysates were analysed by RT-PCR (upper panels) or immunoblotting (lower panels). (**b**–**d**) HEK293 cells were first transfected with siSDC4-2 or siNC. Forty-eight hours later, the cells were co-transfected with RIG-I(N) expression vector and IFN-β (**b**), ISRE (**c**) or NF-κB (**d**) reporter. Twenty hours after the second transfection, the cells were lysed for luciferase assays (upper panel) and immunoblotting assays (lower panels). (**e**–**g**) HEK293 cells were first transfected with siSDC4-1, siSDC4-2 or siNC. Twenty-four hours later, the cells were transfected with IFN-β (**e**), ISRE (**f**) or NF-κB (**g**) reporter. Twenty-four hours after the second transfection, the cells were further infected with SeV for 12 h, and lysed for luciferase assays. (**h**) HEK293 cells were first transfected with siSDC4-3 or siNC. Twenty-four hours later, the cells were transfected with the indicated plasmids. Twenty-four hours after the second transfection, the cells were infected with SeV for 12 h, and lysed for luciferase assays. (**i**,**j**) HEK293 cells were transfected with siSDC4-2 or siNC. Thirty-six hours after transfection, the cells were infected with SeV for 9 h, and then lysed to isolate RNA to measure the transcript levels of *Ifnb1* (**i**) and *Rantes* (**j**) by qRT-PCR. (**k**) HeLa cells were transfected and infected as in (**i**,**j**), then stained with an anti-IRF3 antibody and DAPI, and imaged by confocal microscopy. Bottom panels were the larger magnification. Scale bar, 50 μM. (**l**) HEK293 cells were transfected with siSDC4-2 or siNC. Forty-eight hours later, the cells were infected with SeV for 10 h. The cell lysates were separated by native gel electrophoresis (upper panel) or SDS–PAGE (lower panels) and analysed by immunoblotting with the indicated antibodies. The data in **b**–**j** are from one representative experiment of at least three independent experiments (mean±s.d. of duplicate experiments in **b**–**h** or triplicate experiments in **i** and **j**). A two-tailed Student's *t*-test was used to analyse statistical significance. *P<0.05; **P<0.01; ***P<0.001 versus the control groups.

To better understand SDC4 function, we determined whether knockdown of endogenous SDC4 affects RIG-I-mediated antiviral signalling using three different small interfering RNA (siRNA) oligonucleotides, siSDC4-1, siSDC4-2 and siSDC4-3. Among these, siSDC4-1 and siSDC4-2 were designed to specifically target non-overlapping regions in the coding frame of *Sdc4*, whereas siSDC4-3 targets an untranslated region of the *Sdc4* mRNA. As shown in Fig. 3a, all three siRNAs efficiently reduced the expression of endogenous SDC4 at both mRNA and protein levels in HEK293 cells. Because siSDC4-2 exhibited the highest *Sdc4* knockdown efficiency, we mainly used it in subsequent experiments. Next, we assessed the effect of Sdc4 knockdown on innate immune signalling by employing IFN-β, ISRE and NF-κB luciferase reporter assays as mentioned above. As shown in Fig. 3b–g, compared with the control, knockdown of *Sdc4* significantly enhanced the activation of promoters following RIG-I(N) expression or SeV infection. To validate the specificity of SDC4 function in regulating the RIG-I-mediated signalling, we performed rescue experiments by exogenously introducing SDC4. As shown in Fig. 3h, while knockdown of SDC4 significantly increased the IFN-β luciferase activity induced by SeV infection, when the siSDC4-3, which targets the 3′ untranslated region in *Sdc4*, was used, ectopic expression of the Myc-tagged SDC4 into *Sdc4* knockdown cells almost fully rescued the effects of RNA interference (RNAi). Next, we examined whether alteration of SDC4 expression has an effect on the abundance of mRNAs encoding *Ifnb1* and IFN-stimulated cytokine, such as *Rantes*, and found that knockdown of SDC4 indeed increased the levels of these mRNAs (Fig. 3i,j). We next tested whether *Sdc4* knockdown affects the dimerization and nuclear translocation of IRF3, and found that *Sdc4* knockdown enhanced the nuclear translocation and dimerization of IRF3 following SeV infection (Fig. 3k,l; Supplementary Fig. 6a). In support of these observations, we found that knockout of SDC4 dramatically enhanced the mRNA levels of *Ifnb1* and *Rantes* (Supplementary Fig. 6b–d). Collectively, these results suggest that *Sdc4* inhibits the RIG-I signalling in cultured cells.

### A feedback loop of SDC4–RIG-I regulates viral replication

**Figure 4:**
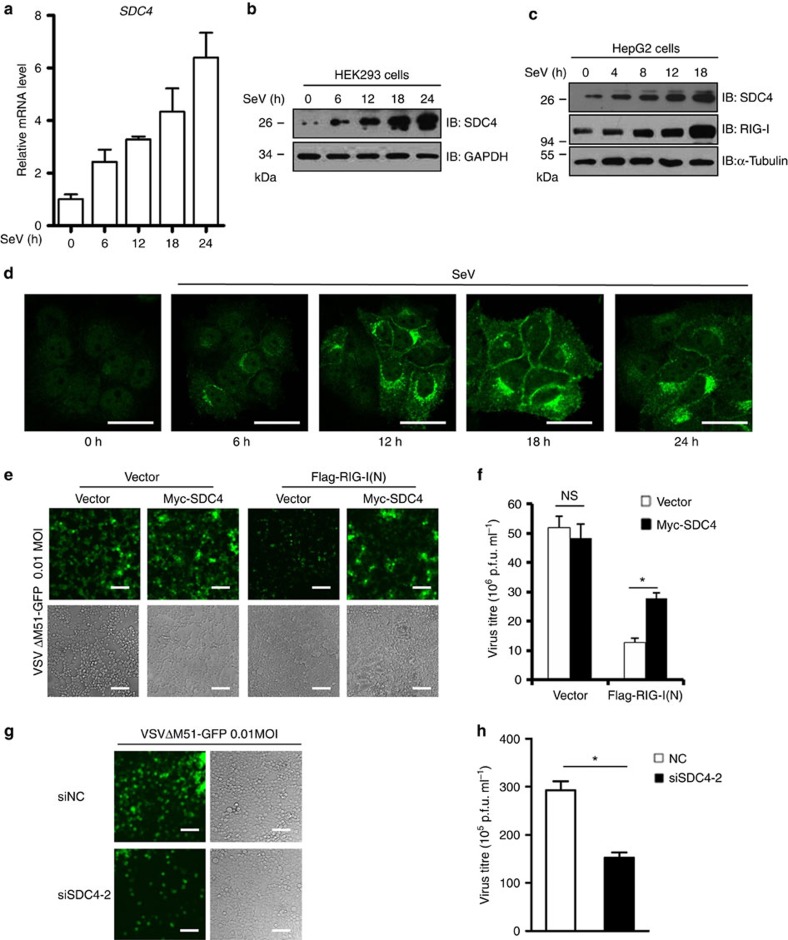
SDC4 is induced by virus infection and involved in regulating viral replication. (**a**) HEK293 cells were infected with SeV for 6, 12, 18 or 24 h, followed by measurements of *Sdc4* mRNA by quantitative real-time PCR analysis. (**b**,**c**) HEK293 cells (**b**) and HepG2 cells (**c**) were infected with SeV for different time courses, and analysed by immunoblotting. (**d**) HeLa cells were infected with SeV for 6, 12, 18 or 24 h, and then fixed with 4% paraformaldehyde, stained with an anti-SDC4 antibody, and observed by confocal microscopy. Scale bar, 50 μM. (**e**,**f**) HEK293 cells were transfected with the indicated expression vectors. Twenty-four hours later, the cells were infected with VSVΔM51-GFP at a MOI of 0.01 for 12 h. Subsequently, the cells were imaged by fluorescence microscopy (**e**), scale bar, 200 μM, or the culture supernatants were collected to measure the virus titre by a plaque assay (**f**). (**g**,**h**) HEK293 cells were transfected with siNC or siSDC4-2. Forty-eight hours after transfection, the cells were infected with VSVΔM51-GFP at a MOI of 0.01 for 12 h. Subsequently, the cells were imaged by fluorescence microscopy (**g**), scale bar, 200 μM, or the culture supernatants were collected to measure the virus titre by a plaque assay (**h**). The data in **a**,**f** and **h** are from one representative experiment of at least three independent experiments (mean±s.d. of triplicate assays). *P<0.05; NS, not significant versus the control groups.

Given that SDC4 has a negative role in controlling RIG-I activity, we attempted to understand the molecular basis of SDC4 regulation. To do so, we measured the dynamics of *Sdc4* mRNA and SDC4 protein expression on virus infection. As shown in Fig. 4a–c, the expression levels of *Sdc4* mRNA and SDC4 protein were significantly increased in a time-dependent manner on virus infection. To confirm this, we performed immunostaining assays using an antibody against endogenous SDC4, and found that while only a weak or non-detectable SDC4 signal was found in HeLa cells under normal conditions, a stronger SDC4 signal was observed when cells were infected with SeV (Fig. 4d). Notably, we found that the induced endogenous SDC4 by viral infection is mainly overlapped with TGN-GFP marker (TGN: the trans-Golgi network) and accumulated in perinuclear region, suggesting that the perinuclear-localized SDC4 is *de novo* synthesized (Supplementary Fig. 7a). Previous study showed that p65 plays an important role in regulating SDC4 expression through two p65-binding sites in the *SDC4* promoter^31^. We next knocked down p65 or IRF3 by siRNA, and found that knockdown of either p65 or IRF3 significantly inhibited SDC4 upregulation induced by SeV infection (Supplementary Fig. 7b–e). Collectively, these findings suggest that SDC4 expression is stimulated by viral infection in a feedback manner.

To further determine whether the reduced type I IFN response mediated by SDC4 is correlated with antiviral immunity, we transfected an expression vector encoding SDC4 or an empty vector, with or without Flag-RIG-I(N), into HEK293 cells. Then, we infected the cells with vesicular stomatitis mutant virus (VSVΔM51–GFP), with a single amino acid deletion (methionine 51) in the matrix (M) protein, at a multiplicity of infection (MOI) of 0.01, and monitored viral replication based on GFP expression and a viral plaque assay. As shown in Fig. 4e,f, Flag-RIG-I(N) overexpression inhibited VSVΔM51-GFP replication, which is consistent with its antiviral role, whereas co-expression with SDC4 rendered the cells more susceptible to viral infection, suggesting that SDC4 negatively regulates the RIG-I-mediated antiviral signalling pathway. However, SDC4 expression did not increase the virus titre compared with that of an empty vector. A possible explanation for this result is that SDC4 plays a role after antiviral signalling was activated. Taken together, these results indicate that the ectopic expression of SDC4 inhibits the RIG-I-mediated type I IFN response, and thus attenuates antiviral immunity. Consistent with this observation, a viral plaque assay and a microscope observation showed that the knockdown of *Sdc4* in HEK293 cells resulted in higher resistance to viral infection and a lower viral titre in the culture supernatants compared with the supernatants of control cells treated with a control siRNA (Fig. 4g,h). Taken together, these results suggest that *Sdc4* knockdown markedly enhances the type I IFN response and antiviral immunity.

### An enhanced type I IFN response in *Sdc4*-deficient cell**s**

**Figure 5:**
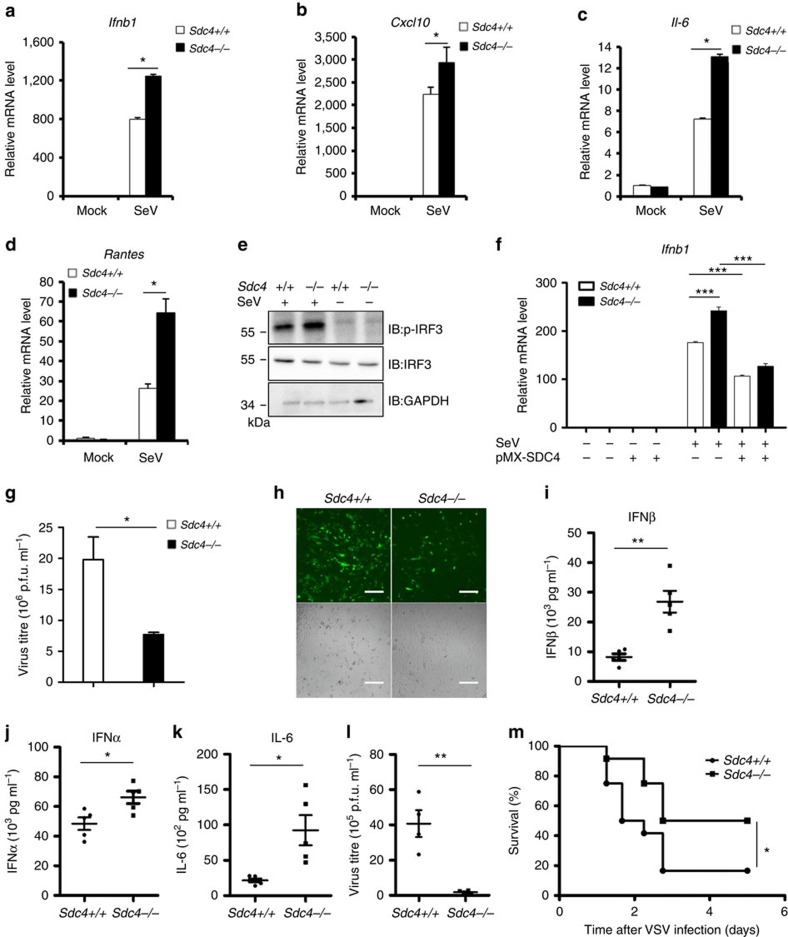
Enhanced type I IFN response in *Sdc4*-deficient cells. (**a**–**d**) Primary *Sdc4*^*+/+*^ and *Sdc4*^–/–^ MEFs were infected with SeV for 6 h, and analysed by quantitative real-time PCR (qRT-PCR). The transcript levels of *Ifnb1* (**a**), *Cxcl10* (b), *Il6* (**c**) and *Rantes* (**d**) were measured by qRT-PCR analysis. (**e**) Primary *Sdc4*^+/+^ and *Sdc4*^*−/−*^ MEFs were left untreated or infected with SeV for 6 h. The cell lysates were separated by SDS–PAGE and analysed by immunoblotting with the indicated antibodies. (**f**) Primary *Sdc4*^*+/+*^ and *Sdc4*^*−/−*^ MEFs were first infected with a retrovirus expressing SDC4 or an empty vector. After 48 h of infection, the cells were infected with SeV for 7 h, and analysed by qRT-PCR. (**g**,**h**) Primary *Sdc4*^*+/+*^ and *Sdc4*^*−/−*^ MEFs were infected with VSVΔM51-GFP at a MOI of 0.01 for 22 h. Subsequently, the culture supernatants were collected to measure the virus titre by a plaque assay (**g**) or the cells were imaged by fluorescence microscopy (**h**), scale bar, 200 μM. (**i**–**k**) *Sdc4*^+/+^ and *Sdc4*^*−/−*^ mice (*n*=5 each) were infected with VSVΔM51-GFP via tail vein injection at 2 × 10^8^ p.f.u. per mouse. Sera were collected after 6 h infection to measure the levels of IFN-β (**i**), IFN-α (**j**) and IL-6 (**k**) by ELISA. (**l**) *Sdc4*^+/+^ and *Sdc4*^*−/−*^ mice (*n*=5 each) were infected with VSVΔM51-GFP via tail vein injection at 1 × 10^9^ p.f.u. per mouse. Sera were collected after 12 h infection to measure the virus titre by a plaque assay. (**m**) *Sdc4*^+/+^ and *Sdc4*^*−/−*^ mice (*n*=14 each) were infected with VSV virus via tail vein injection at 4 × 10^8^ p.f.u. per mouse and the survival of mice were monitored for 1 week. The data in **a**–**d**, **f**–**g** and **i**–**l** are from one representative experiment of at least three independent experiments (mean±s.d. of triplicate experiments in **a**–**d** and **f**, duplicate experiments in **g**). A two-tailed Student's *t*-test or log-rank (Mantel–Cox) test were used to analyse statistical significance. *P<0.05; **P<0.01; ***P<0.001 versus the control groups.

To substantiate our findings, we generated *Sdc4* knockout mice using the CRISPR/Cas9 system^32^,^33^, the mutant mice were born at the Mendelian ratio, fertile, and developed normally (Supplementary Fig. 8a,b and Supplementary Tables 1 and 2). To determine whether the Sdc4 knockout affects the type I IFN response after virus infection, we infected Sdc4^–/–^ mouse embryonic fibroblasts (MEFs) with SeV and measured the expression levels of type I IFN-dependent genes. As shown in Fig. 5a–d, knockout of *Sdc4* resulted in a significant increase in mRNA levels of *Ifnb1* or IFN-stimulated cytokine genes, such as *Cxcl10*, *Il6* and *Rantes*, when compared with the wild-type control. Furthermore, we observed that the *Sdc4* knockout also increased the abundance of *Ifnb1*, *Cxcl10* and *Il6* transcripts in mouse bone marrow-derived macrophages (Supplementary Fig. 8c–e). In addition, the phosphorylation of IRF3 was increased in *Sdc4*^−/−^ MEF cells compared with that in *Sdc4*^+/+^ MEF cells (Fig. 5e). To determine the specific role of SDC4 in antiviral signalling, we performed a rescue experiment via the retroviral expression of mouse *Sdc4* in *Sdc4*^−/−^ MEF cells. As shown in Fig. 5f, retroviral expressing mouse *Sdc4* apparently reversed the SeV-mediated enhancement of *Ifnb1* expression in *Sdc4*^–/–^ MEFs. These observations indicate that SDC4 plays a negative role in regulating the antiviral signalling.

To further understand the biological function of SDC4, we performed viral replication assays using *Sdc4*^*−/−*^ MEF cells. As shown in Fig. 5g,h, a lower titre of VSVΔM51-GFP was produced in *Sdc4*^*−/−*^ MEF cells than in wild-type control cells. Consistently, we found that much fewer GFP-positive cells were observed in *Sdc4*^*−/−*^ MEF when compared with the wild-type control in the microscopy assay. These results provide additional biological evidence that SDC4 is a negative regulator of IRF3 activation, IFN-β production and cellular antiviral immunity.

### SDC4 negatively regulates immune responses *in vivo*

To examine the role of SDC4 in regulating the immune defence against RNA virus infection *in vivo*, we infected *Sdc4*^+/+^ and *Sdc4*^*−/−*^ mice with VSVΔM51-GFP virus via the intravenous route, and then measured the levels of IFN-β, IFN-α and IL-6 in the sera of infected mice by enzyme-linked immunosorbent assays (ELISAs). As shown in Fig. 5i–k, the induction of these cytokines was significantly higher in *Sdc4*^*−/−*^mice than in wild-type control mice. In line with these observations, a lower VSVΔM51-GFP titre was detected in *Sdc4*^*−/−*^mice than in wild-type control mice (Fig. 5l). Furthermore, we carried out the survival rate assays, and found that SDC4-deficient mice were more resistant to virus infection, compared with the wild-type control (Fig. 5m). These results provide *in vivo* evidence that SDC4 plays an important role in antiviral innate immunity.

### SDC4 decreases the K63-linkage ubiquitination of RIG-I

The aforementioned experiments show that SDC4 specially inhibits the RIG-I(N)-induced type I IFN response, whereas it does not influence the activity of MDA5(N). Therefore, we subsequently focused on investigating the mechanism by which SDC4 regulates RIG-I activation. A number of studies have suggested that ubiquitin modifications of RIG-I play important roles in either activating or fine-tuning activity of RIG-I during antiviral signal transduction^6^,^11^,^13^,^14^,^15^. K48-linked ubiquitination of RIG-I negatively regulates the type I IFN response through the proteasome-mediated degradation of RIG-I (ref. 12); however, K63-linked ubiquitination of RIG-I(N) is essential for its activation and triggering the production of type I IFNs^14^,^27^.

**Figure 6:**
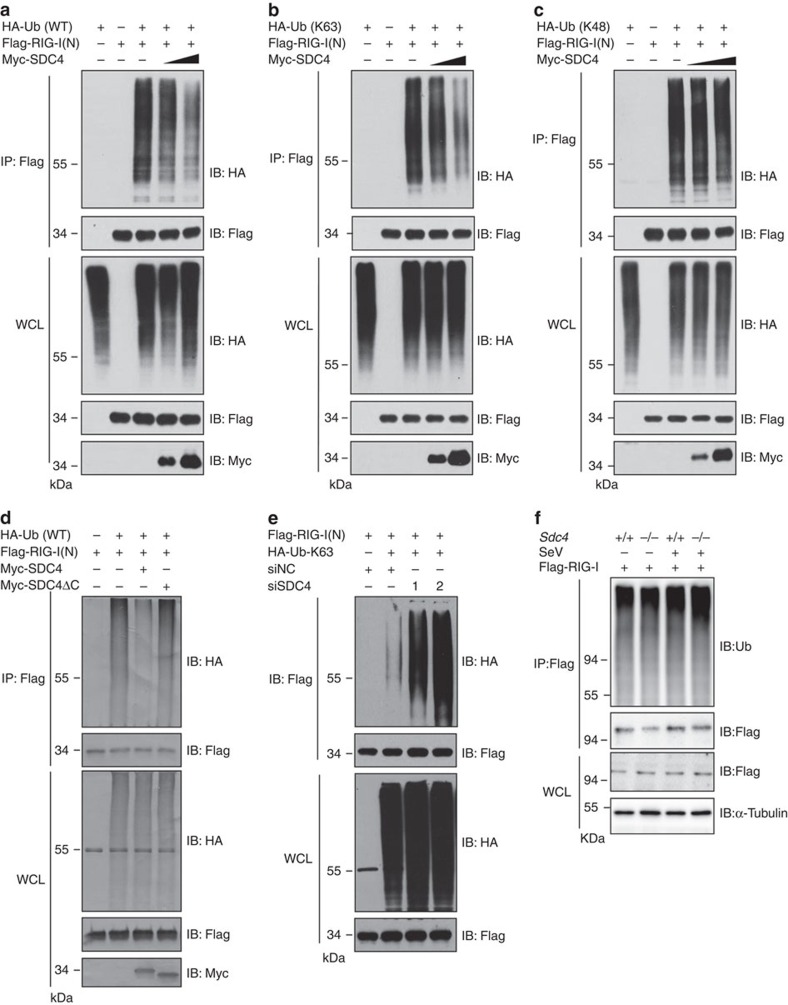
SDC4 reduces the K63-linkage ubiquitination of RIG-I. (**a**–**c**) HEK293 cells were transfected with SDC4 and RIG-I(N) together with HA-tagged wild-type Ub (HA-Ub-WT) (**a**), HA-Ub-K63 (**b**) or HA-Ub-K48 (**c**) plasmids. Twenty-four hours after transfection, cell lysates were immunoprecipitated with anti-Flag beads, and subjected to immunoblotting analysis with the indicated antibodies. The expression levels of transfected proteins in WCL are shown in the bottom panels. (**d**) HEK293 cells were transfected with Flag-RIG-I(N) and HA-Ub-WT together with the Myc-SDC4 or Myc-SDC4-ΔC plasmids. Twenty-four hours after transfection, cell lysates were immunoprecipitated with anti-Flag beads, and subjected to immunoblotting analysis with the indicated antibodies. The expression levels of transfected proteins in WCL are shown in the bottom panels. (**e**) HEK293 cells were first transfected with siNC or siSDC4-2. Twenty-four hours after transfection, cells were transfected with the indicated combinations of expression plasmids. Twenty-four hours after the second transfection, cell lysates were immunoprecipitated with anti-Flag beads, and analysed by immunoblotting with the indicated antibodies. (**f**) Primary *Sdc4*^+/+^ and *Sdc4*^*−/−*^ MEFs were first infected with a retrovirus expressing mouse RIG-I for 48 h. The cells were infected with SeV (100 HA units per ml) for 6 h. Subsequently, cell lysates were immunoprecipitated with anti-Flag beads and analysed by immunoblotting with the indicated antibodies. The data shown in **a**–**f** are from one representative experiment of at least three independent experiments.

To test whether SDC4 regulates RIG-I function by affecting RIG-I ubiquitination, we performed *in vivo* ubiquitination assays in HEK293 cells. As shown in Fig. 6a, RIG-I(N) was polyubiquitinated when cells were co-transfected with a vector expressing wild-type ubiquitin, while the ubiquitination of RIG-I(N) was abrogated in a dose-dependent manner by SDC4 co-expression, suggesting that SDC4 plays a specific role in regulating the ubiquitination of RIG-I. To distinguish which type of lysine linkage (K63 or K48) of RIG-I(N) is regulated by SDC4, we employed a ubiquitin mutant in which only one lysine (at position K63 or K48) was available for ubiquitination. As shown in Fig. 6b,c and Supplementary Fig. 9, the K63-linked ubiquitination of RIG-I(N) was markedly decreased by co-expression of SDC4, whereas K63-linked ubiquitination of RIG-I(RD) and the K48-linked ubiquitination of RIG-I(N) were not influenced under the same conditions. These findings suggest that SDC4 regulates K63-linked ubiquitination of RIG-I at N-terminal region. Consistent with reporter results, SDC4-ΔC did not affect the ubiquitination of RIG-I(N) (Fig. 6d), indicating that the CP domain of SDC4 is essential for its inhibitory function. To confirm our observation, we tested the level of RIG-I(N) ubiquitination after *Sdc4* knockdown or knockout. As expected, knockdown of endogenous *Sdc4* enhanced the K63-linked ubiquitination of RIG-I(N) in HEK293 cells (Fig. 6e). Moreover, the SeV-mediated ubiquitination of RIG-I was significantly increased in *Sdc4*^*−/−*^MEF cells (Fig. 6f). Taken together, our findings support the notion that SDC4 negatively modulates the K63-linked ubiquitination of RIG-I to suppress the type I IFN response.

### SDC4 interacts with and recruits CYLD to the RIG-I complex

**Figure 7:**
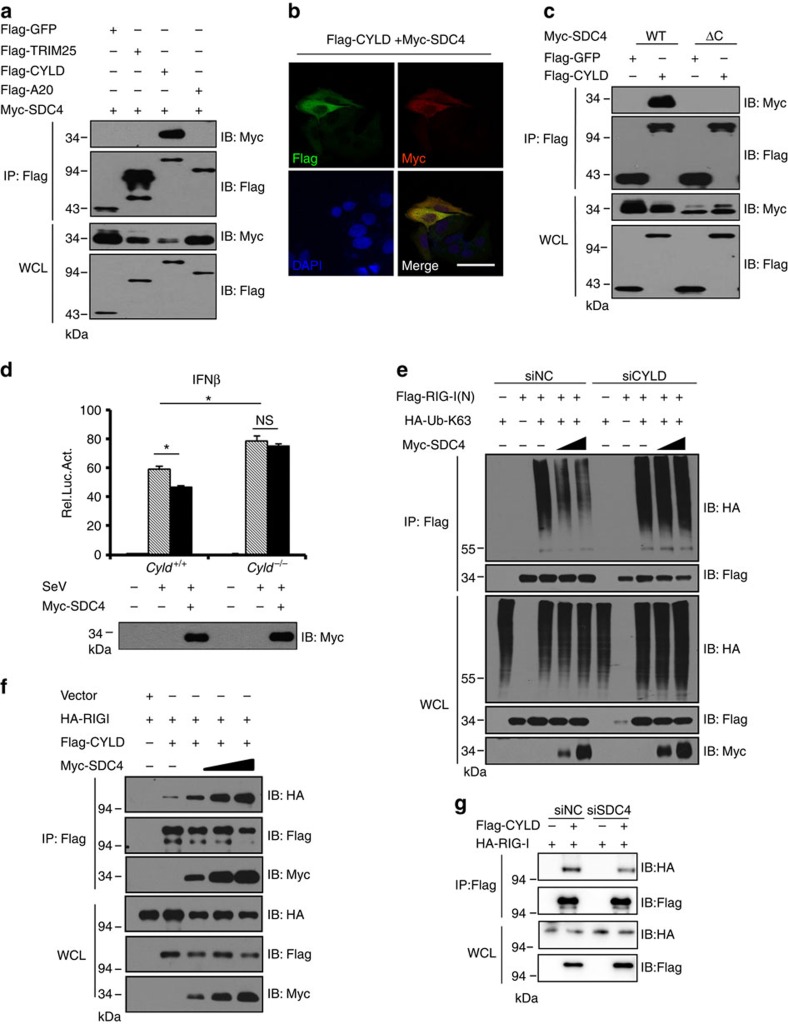
SDC4 interacts with and recruits CYLD to the RIG-I complex. (**a**) HEK293 cells were transfected with the indicated expression plasmids. Twenty-four hours post transfection, the lysates were immunoprecipitated with anti-Flag beads, and analysed by immunoblotting with the indicated antibodies. Bottom panels, expression of exogenous proteins in WCL. (**b**) HeLa cells were transfected with the indicated plasmids. Twenty-four hours after transfection, the cells were fixed, stained with the indicated antibodies, and observed by confocal microscopy. Scale bar, 50 μM. (**c**) HEK293 cells were transfected with the indicated expression plasmids. Twenty-four hours post transfection, the lysates were immunoprecipitated with anti-Flag beads, and analysed by immunoblotting with the indicated antibodies. (**d**) A *Cyld*^*−/−*^ stable cell line was generated by the CRISPR/Cas9-mediated gene editing system. *Cyld*^*−/−*^ and control cells (Cas9-vector) were co-transfected with an empty vector or Myc-SDC4 and the indicated reporter plasmids. Forty-eight hours after transfection, the cells were left untreated or infected with SeV (50 HA units per ml) for 16 h, and analysed by luciferase assays (upper panel) and immunoblotting assays (lower panel). (**e**) HEK293 cells were first transfected with siNC or siCYLD. Twenty-four hours post transfection, the cells were transfected with the indicated combinations of expression plasmids. Twenty-four hours after the second transfection, cell lysates were immunoprecipitated with anti-Flag beads, and analysed by immunoblotting with the indicated antibodies. (**f**) HEK293 cells were transfected with the indicated expression plasmids. Twenty-four hours post transfection, the lysates were immunoprecipitated with anti-Flag beads, and analysed by immunoblotting with the indicated antibodies. (**g**) HEK293 cells were first transfected with siNC or siSDC4. Twenty-four hours post transfection, the cells were transfected with the indicated combinations of expression plasmids. Twenty-four hours after the second transfection, cell lysates were immunoprecipitated with anti-Flag beads, and analysed by immunoblotting with the indicated antibodies. The data in **d** are from one representative experiment of at least three independent experiments (mean±s.d. of duplicate assays). A two-tailed Student's *t*-test was used to analyse statistical significance. *P<0.05; NS, not significant versus the control groups.

Next, we made an effort to understand the mechanism by which SDC4 modulated the K63-linked ubiquitination of RIG-I(N). Over the past several years, the regulation of RIG-I K63-linked ubiquitin chains has been particularly well characterized^6^,^11^. Two well-studied ubiquitin ligase and deubiquitinase are TRIM25 (ref. 6) and CYLD^13^, respectively, which are responsible for RIG-I K63-linked ubiquitin chain conjugation and deconjugation, respectively. To test whether SDC4 regulates RIG-I ubiquitination through TRIM25 or CYLD, we first tested whether SDC4 could form a complex with TRIM25 or CYLD. As shown in the co-IP assays (Fig. 7a), CYLD, but not TRIM25, was present in the SDC4 immunoprecipitate, suggesting a strong association of SDC4 with the deubiquitinating enzyme CYLD, rather than the ubiquitin E3 ligase TRIM25. Importantly, we noted that the SDC4-CYLD association is specific, as no apparent association between SDC4 and A20, another deubiquitinating enzyme that is involved in regulating antiviral signalling^34^, was observed in transfected cells. Consistently, endogenous CYLD was detected in the SDC4 complex in the absence of viral infection (Supplementary Fig. 2). Interestingly, the association between CYLD and SDC4 was significantly enhanced after the cells were infected with Sendai virus (Supplementary Fig. 2). To confirm this observation, we transfected epitope-tagged SDC4 and CYLD into HeLa cells, and a confocal microscopy assay showed that SDC4 was co-localized with CYLD in HeLa cells (Fig. 7b). Interestingly, SDC4ΔC was also unable to bind CYLD (Fig. 7c), which indicates that the CP region of SDC4 is necessary for its interaction with either RIG-I or CYLD. To identify which domain of CYLD is responsible for the SDC4 interaction, we generated a series of mutants as indicated in Supplementary Fig. 10a, and performed co-IP assays in HEK293 cells. As shown in Supplementary Fig. 10b, the mutant CYLD-(M1) (deleted for amino acids 556–655) abrogated the association with SDC4, suggesting that amino acids 556–655 of CYLD are required for its interaction with SDC4. Because CYLD is a deubiquitinating enzyme, and amino acids 556–655 include the catalytic active site residue C601, we wanted to know whether CYLD enzymatic activity was required for its interaction with SDC4. As shown in Supplementary Fig. 10c, both the wild-type and catalytically inactive mutant proteins were present in the SDC4 immunoprecipitate, suggesting that the association between SDC4 and CYLD is independent of the enzymatic activity of CYLD. On the basis of these results, we speculate that SDC4 likely regulates RIG-I ubiquitination in a CYLD-dependent manner. To test this hypothesis, we first knocked out CYLD in HEK293 cells and determined whether the SDC4 inhibitory function was affected. As shown in Fig. 7d, compared with control cells, the CYLD knockout significantly inhibited the SDC4-mediated inhibition of IFN-β activity in response to SeV infection. To better understand the relationship between SDC4 and CYLD, we performed additional ubiquitination assays in HEK293 cells. As shown in Fig. 7e and Supplementary Fig. 11a, SDC4 progressively reduced the ubiquitination of RIG-I(N) in a dose-dependent manner; however, RIG-I(N) was apparently resistant to deubiquitination mediated by SDC4 when CYLD expression was knocked down by RNAi in transfected cells. Similar results were obtained when CYLD was knocked out in HEK293 cells (Supplementary Fig. 11b). These findings suggest that SDC4 regulates RIG-I deubiquitination in a CYLD-dependent manner.

To further dissect how SDC4 regulates RIG-I deubiquitination through CYLD, we first examined the cellular location of SDC4, RIG-I and CYLD after virus infection, as a previous study has shown that the redistribution of RIG-I to the membrane in a perinuclear pattern plays an important role in antiviral signal transduction^16^. As shown in Supplementary Fig. 12a, similar to the sub-cellular pattern of RIG-I, SDC4 and CYLD were also accumulated in a perinuclear pattern on SeV infection, raising a possibility that SDC4 has a potential role in affecting the RIG-I–CYLD interaction and their perinuclear localization as well. Several lines of evidence support this idea. First, our co-IP experiments showed that SDC4 overexpression indeed enhanced the interaction between CYLD and RIG-I, whereas SDC4 knockdown reduced the association of CYLD with RIG-I (Fig. 7f,g). Second, the effect mediated by SDC4 is specific on the RIG-I–CYLD interaction, because the RIG-I-TRIM25 association was not affected by SDC4 (Supplementary Fig. 12b). Third, knockdown of SDC4 apparently abolished the perinuclear localization of RIG-I and CYLD (Supplementary Fig. 12c). In addition, membrane fraction assays showed that SDC4, RIG-I and CYLD accumulated at membrane fraction on virus infection (Supplementary Fig. 12d). Taken together, these results suggest that SDC4 enhances the interaction between RIG-I and CYLD and their perinuclear localization, thereby regulating RIG-I deubiquitination in a CYLD-dependent manner.

## Discussion

RIG-I is an important receptor for viral RNAs, and it is activated on virus infection. However, its activation must be under a tight regulation, otherwise aberrant immune responses may occur, leading to autoimmune or chronic inflammatory diseases. In this study, we demonstrated that SDC4, a heparan sulfate proteoglycan, physically interacts with RIG-I and negatively regulates antiviral signalling in human and mouse cells. Moreover, we found that SDC4 uses its carboxyl-terminal fragment to recruit CYLD to the RIG-I complex, and decreases K63-linked ubiquitination in a CYLD-dependent manner. Thus, our study has established a novel regulatory mechanism by which SDC4 regulates viral replication and the type I IFN response.

Negative regulatory factors usually function as negative feedback molecules to attenuate responses. Because SDC4 plays a negative role in the regulation of antiviral signalling, we sought to determine the molecular basis of SDC4 action in the antiviral signalling. To do so, we measured the dynamics of *Sdc4* mRNA and SDC4 protein expression on virus infection. As shown in Fig. 4a–c, coincident with enhanced IRF3 signalling and IFN-β production, *Sdc4* mRNA and SDC4 protein were significantly increased in HEK293 cells and HepG2 cells on virus infection. To confirm this, we performed immunostaining assays using an antibody against endogenous SDC4. As shown in Fig. 4d, we found that while only a weak or non-detectable SDC4 signal was found in cells under normal conditions, a stronger SDC4 signal was accumulated in a perinuclear pattern after cells were infected with SeV. These findings suggest that SDC4 might be induced as part of a feedback regulatory mechanism to suppress IFN-β production during viral infection.

On the basis of our results, we propose the following working model to illustrate how SDC4 could negatively regulate RIG-I-mediated type I IFN signalling on viral infection. First, the carboxyl-terminal RNA helicase domain of RIG-I binds to viral RNA, which results in a conformational change in RIG-I. Then, the amino-terminal CARD domains of RIG-I are exposed and undergo K63-linked ubiquitination by an E3 ligase, such as TRIM25, thereby allowing the activation of RIG-I. Then, activated RIG-I physically interacts with the adaptor protein MAVS to trigger downstream signalling. Meanwhile, the mRNA transcript and protein levels of SDC4 are increased on virus infection. Newly synthesized SDC4 then recruits CYLD and RIG-I to form a large complex in the membrane compartment in a perinuclear pattern, which assists the interaction between CYLD and RIG-I, as well as the deubiquitination of the K63-linked ubiquitin of RIG-I. Importantly, we found that a carboxyl-terminal fragment of SDC4 containing only 28 amino-acid residues was essential for the inhibitory function of SDC4. A simple explanation for this result is that the carboxyl-terminal fragment of SDC4 brings RIG-I and CYLD close together, which promotes the deubiquitination of RIG-I by CYLD. Previous studies suggest that in addition to RIG-I, MDA5 also functions as a receptor in anti-viral signalling^3^, raising a possibility of whether the SDC4/CYLD targets to MDA5. Our co-IP analysis revealed that CYLD was not present in the MDA5 immunoprecipitants (Supplementary Fig. 4c,d), and suggest that MDA5 is not the primary target of the SDC4/CYLD deubiquitinase complex.

In conclusion, we identified a previously unrecognized role of SDC4 in regulating RIG-I-mediated antiviral signalling. SDC4 specifically interacted with RIG-I and CYLD and decreased the K63-linked ubiquitination of RIG-I to prevent the excessive production of type I IFN. Our data provide information on the mechanisms by which SDC4 negatively regulates the type I IFN response to help balance innate immunity and immune tolerance.

Of note, our finding that SDC4 strongly interacts with CYLD is very intriguing. Given SDC4 also plays important roles in regulating cell migration^20^,^21^,^22^, adhesion^19^ and angiogenesis^25^; and is strongly associated with arthritis^22^, asthma induction^21^, inflammation^35^ and cancer^36^. It will be important to investigate whether CYLD is also involved in other functions of SDC4 regulation in the future.

## Methods

### Ethics statements

All animal studies were conducted in strict accordance with the recommendations in the Guide for the Care and Use of Laboratory Animals of the Ministry of Science and Technology of the People's Republic of China. The protocols for animal studies were approved by the Committee on the Ethics of Animal Experiments of the Institute of Zoology, Chinese Academy of Sciences (approval number: IOZ15001).

### Generation of *Sdc4* knockout mice

To generate *Sdc4* knockout mice, the CRISPR/Cas9-mediated gene deletion system was used. Cas9 mRNA and single-guide RNAs targeting different *Sdc4* sequences were co-injected into zygotes to obtain heterozygous mutants. Homozygous mice were obtained by breeding heterozygote mutants. The sequences targeting *Sdc4* were (5′–3′): GCAGCAGCAGCGGCGCAAGC and AGCATCTTCGTCGTCGGGGA . Three kinds of mutations were obtained: −1 bp, −10 bp and +1 bp deletions or insertion in the first exon of *Sdc4*, all of which are frameshift mutations that prematurely terminate protein translation. *Sdc4* knockout mice was verified by DNA sequences and immunoblotting analysis (Supplementary Fig. 8a,b)

### Cell culture and viruses

HEK293, HeLa, HepG2 and Vero cells were obtained from the Shanghai Cell Bank of the Chinese Academy of Sciences, and maintained in Dulbecco's modified Eagle's medium (DMEM) supplemented with 10% fetal bovine serum, 1% penicillin and 1% streptomycin. *Sdc4*^+/+^ and *Sdc4*^–/–^ MEFs were generated from 13.5-day-old embryos and maintained in complete DMEM containing 1 mM sodium pyruvate, 10 μM L-glutamine, 10 μM β-mercaptoethanol and 1% nonessential amino acids. bone marrow-derived macrophages were prepared as previously described^37^. Briefly, bone marrow cells were collected from femurs and tibiae of mice, and cultured in complete DMEM containing conditioned media from a 5-day culture of L929 cells for 5 days, and then the cells were collected for experiments. SeV, VSV (Indiana strain) and VSVΔM51-GFP have been described previously^38^. For virus plaque assay, confluent Vero cells were used for infection with the diluted virus of VSVΔM51-GFP or VSV for 1 h. Culture medium containing 2% methylcellulose was overlaid and incubated for about 36 h. The cells were then fixed for 15 min with methanol and stained with 1% crystal violet to display plaques. Plaques were counted to quantitate the viral titre as plaque-forming units per ml.

### Plasmids

Mammalian expression plasmids encoding Myc-tagged SDC1, SDC2, SDC3 and SDC4, HA-tagged RIG-I, Ub, K63-Ub and K48-Ub, and Flag-tagged RIG-I, RIG-I(N), RIG-I(RD), SDC4, TRIM25, A20 and CYLD were constructed with standard molecular biology techniques. Various mutants, including SDC4 mutants and CYLD mutants, were generated by PCR via the internal deletion method. NF-κB-luc reporter was generously provided by Dr Zhijian Chen, IFN-β-luc and ISRE-luc plasmids were kindly provided by Dr Hongbing Shu^39^.

### Transfection and luciferase reporter analysis

HEK293 cells were seeded in 24-well plates at a density of 1.0 × 10^5^ cells per well and transfected the following day via the standard calcium phosphate transfection method. A pRSV/LacZ reporter plasmid (50 ng) and a firefly luciferase reporter plasmid (50 ng) were co-transfected with the indicated expression plasmids. In the same experiment, an empty control plasmid was added to ensure that the same amount of total DNA was transfected. Luciferase activity was measured at the indicated time points and normalized to the LacZ activity. All reporter assays were repeated at least three times.

### RNAi

HEK293 cells were transfected with siRNAs at a final concentration of 40 nM via the standard calcium phosphate transfection method. After the indicated time, the cells were transfected with the indicated plasmids using Lipofectamine 2000 (Invitrogen) or stimulated with virus, and then collected for subsequent reporter, WB, or real-time RCR analysis. The siRNA sequences were as follows (5′–3′; only the sense strand is shown): NC (non-targeting), UUCUCCGAACGUGUCACGU ; SDC4-1, GUUGUCCAUCCCUUGGUGC ; SDC4-2, GCAAGAAACCCAUCUACAA ; SDC4-3: CAGGUUCUUCUUGAGCUUU ; CYLD-1, CCUCAUGCAGUUCUCUUUG ; CYLD-2 GAUCGUUCUGUGGGGCAUU ; IRF3, GGAGGAUUUCGGAAUCUUC ; p65, GCCCUAUCCCUUUACGUCA .

### Co-immunoprecipitation and immunoblotting analysis

These experiments were performed as the following method. For semi-endogenous co-IP, cells were lysed in 0.5% TritonX-100 lysis buffer (50 mM Tris-Cl at pH 7.5, 150 mM NaCl, 0.5% TritonX-100, 10% glycerol and 1 mM ethylenediaminetetraacetic acid (EDTA)). Cell lysates were incubated with anti-Flag agarose beads (Sigma) for 4–6 h at 4 °C. The immune complexes were washed three times with lysis buffer, and analysed by immunoblotting. For endogenous co-IP, cells were lysed in 0.2% TritonX-100 lysis buffer (20 mM Tris-Cl at pH 7.5, 100 mM NaCl, 0.2% TritonX-100, 10% glycerol and 10 mM EDTA). The cell lysates were incubated with anti-SDC4 antibody or control IgG overnight at 4 °C, and incubated with proteinA/G agarose beads (Thermo) for 2–3 h. The immune complexes were washed three times with lysis buffer and analysed by immunoblotting according to the standard procedures. The following antibodies were used in immunoblotting analysis: anti-SDC4 antibody (PAB9045, 1:1,000) was purchased from Abnova; antibodies for anti-CYLD (8462S, 1:1,000), anti-phospho-IRF3 (4947S, 1:1,000) and anti-IRF3 (4302S, 1:1,000) were from Cell Signaling Technology; anti-IRF3 antibody (sc-33641, 1:1,000 for the IRF3 dimer) was from Santa Cruz Biotechnology; antibodies for anti-GAPDH (KM9002, 1:5,000) and anti-α-Tubulin (KM9007, 1:5,000) were from Sungene Biotechnology; anti-Flag antibody (F7452 1:2,000) was from Sigma; anti-hemagglutinin (HA) (T501, 1:2,000), anti-Ubiquitin (D058-3, 1:1,000) and anti-Myc antibodies (562, 1:2,000) were from MBL. The antibody against human RIG-I was generated by immunizing rabbits with a recombinant glutathione *S*-transferase (GST)-RIG-I (amino acids 1–242) fusion protein that was produced in *Escherichia coli* (*E. coli*) (1:5,000). The antibody against human SDC4 was generated by immunizing mouse with a recombinant GST-hSDC4 (amino acids 1–100) fusion protein that was produced in *E. coli* (1:5,000). The antibody against mouse SDC4 was generated by immunizing rabbit with a recombinant GST-mSDC4 (amino acids 1–100) fusion protein that was produced in *E. coli* (1:2,000). Images have been cropped for presentation. Full-size images are presented in Supplementary Fig. 13.

### Cell immunofluorescence

For microscopy images, HeLa cells were grown on Geltin-coated glass coverslips and transfected or infected as described. After washing with PBS, cells were fixed with 4% paraformaldehyde for 10 min, permeabilized and blocked with 0.2% Triton X-100 in PBS containing 5% BSA for 30 min at room temperature. Cells were then incubated with primary antibody and secondary antibody, respectively. Cells were washed with PBS between each step. Coverslips were mounted and cells were imaged on a Zeiss LSM 510 META analyzer.

### *In vivo* ubiquitination assay

*In vivo* ubiquitination assays were performed by the following method. Briefly, HEK293 cells were transfected with the indicated DNA constructs. Twenty-four hours post transfection, cells were treated with MG132 (final concentration 25 μM) for 4 h. The cells were lysed in 100 μl of lysis buffer A (150 mM NaCl, 50 mM Tris-HCl, pH 7.4, 10% glycerol, 1% Triton X-100, 1% sodium dodecyl sulfate (SDS), and 10 mM *N*-Ethylmaleimide), diluted with 1 ml of buffer B (150 mM NaCl, 50 mM Tris-HCl, pH 7.4, 10% glycerol, 50 mM EDTA and 1% Triton X-100), and sonicated briefly. After incubating with anti-Flag beads for 4 h, the beads were extensively washed three times with lysis buffer containing 0.1% SDS and 500 mM NaCl for 1 h. Samples were then subjected to western blotting analysis with the indicated antibodies.

### Yeast two-hybrid screening

To perform the yeast two-hybrid screening, RIG-I(N) (amino acids 1–242) was inserted into the bait pGBKT7 vector. A human fetal liver complementary DNA library was screened according to the protocol recommended by the manufacturer (Clontech).

### Quantitative real-time PCR

Total RNA was extracted using TRIZOL reagent (Invitrogen). cDNA was synthesized using the SuperScript III First-Strand cDNA Synthesis kit (Invitrogen). Real-time PCR was performed using SYBR Green Master Mix (Thermo Fisher Scientific) in triplicate on a Light Cycler 480 (Roche). Relative levels of mRNA were normalized to the GAPDH RNA levels in each sample. Data shown are the abundance of the mRNA relative to that of control groups. The primers used were as follows (5′–3′):

hGAPDH-S: ATGACATCAAGAAGGTGGTG

hGAPDH-AS: CATACCAGGAAATGAGCTTG

hIFNB1-S: AGGACAGGATGAACTTTGAC

hIFNB1-AS: TGATAGACATTAGCCAGGAG

hRANTES-S: TCCAACCCAGCAGTCGTCT

hRANTES-AS: TTGGCGGTTCTTTCGGGTG

hSDC4-S: CCAACAAGGTGTCAATGTCCAGCA

hSDC4-AS: TGTAGATGGGTTTCTTGCCCAGGT

hIRF3-S: ACAGCAGGAGGATTTCGG

hIRF3-AS: CCCTTCTTTGCGGTTGAG

hp65-S: GGGGACTACGACCTGAATG

hp65-AS: GGGCACGATTGTCAAAGAT

mGAPDH-S: AACTTTGGCATTGTGGAAGG

mGAPDH-AS: ACACATTGGGGGTAGGAACA

mCxcl10-S: GAATCCGGAATCTAAGACCATCAA

mCxcl10-AS: GTGCGTGGCTTCACTCCAGT

mIFNB1-S: ATGGTGGTCCGAGCAGAGAT

mIFNB1-AS: CCACCACTCATTCTGAGGCA

mIL-6-S: TCCATCCAGTTGCCTTCTTG

mIL-6-AS: GGTCTGTTGGGAGTGGTATC

mRANTES-S: CTCACCATATGGCTCGGACA

mRANTES-AS: ACAAACACGACTGCAAGATTGG

### CRISPR9/Cas9-mediated SDC4 or CYLD knockout cell lines

To generate stable HEK293 SDC4 knockout cell lines, HEK293 cells were transfected with the pEPKO or pEPKO-puro-SDC4 vectors. pEPKO-puro-SDC4 vectors were constructed according to the protocol recommended by Cong *et al*.^32^. The sequences targeting SDC4 were (5′–3′): ACCGCAGCGCGAACAGACGGGCGG , ACCGACAACTTCAGGGCCGATCA . To generate stable HEK293 CYLD knockout cell lines, HEK293 cells were transfected with the pEPKO or pEPKO-puro-CYLD vectors, which were kindly provided by Professor Xia Zongping at Zhejiang University. Then, the cells were selected with puromycin (1 μg ml^−1^) for 1 week. The protein levels were confirmed by immunoblotting.

### Cell membrane protein isolation

HeLa cells were infected with or without SeV (50 HA units per ml) for 12 h, the cells were collected and the total membrane protein was isolated using Minute Plasma Membrane protein Isolation Kit (Invent Biotechnologies, SM-005).

### Viral infection in mice and ELISA

Mice of different genotypes were infected with VSV (4 × 10^8^ p.f.u. per mouse) via tail vein injection and the survival of mice was monitored for 1 week. To measure the level of serum cytokines after virus infection *in vivo*, mice of different genotypes were infected with VSVΔM51-GFP (2 × 10^8^  p.f.u. per mouse) via tail vein injection. Sera were collected after 6 h infection to measure the production of IFN-α, IFN-β and IL-6 by ELISA according to the manufacturer's instructions. ELISA kits for mouse IFN-α and IFN-β were purchased from PBL Biomedical Laboratories, and the IL-6 kit was from BD Biosciences.

### Statistical analyses

Log-rank (Mantel–Cox) test was used for survival analysis. The rest statistical analyses are shown as means and standard errors. The significance of the differences between groups was assessed with a two-tailed Student's *t*-test.

### Data availability

The authors declare that the data supporting the findings of this study are available within the article and its Supplementary Information file.

## Additional information

**How to cite this article**: Lin, W. *et al*. Syndecan-4 negatively regulates antiviral signalling by mediating RIG-I deubiquitination via CYLD. *Nat. Commun.* 7:11848 doi: 10.1038/ncomms11848 (2016).

## Supplementary Material

Supplementary Information Supplementary Figures 1 - 13 and Supplementary Tables 1 and 2
