# Supplementary material for: Syndecan-4 negatively regulates antiviral signalling by mediating RIG-I deubiquitination via CYLD
Source: Nat Commun. 2016 Jun 9;7:11848. doi: 10.1038/ncomms11848 (PMC4906230; doi:10.1038/ncomms11848)
Supplement: Supplementary Information — Supplementary Figures 1 - 13 and Supplementary Tables 1 and 2 [file ncomms11848-s1.pdf]

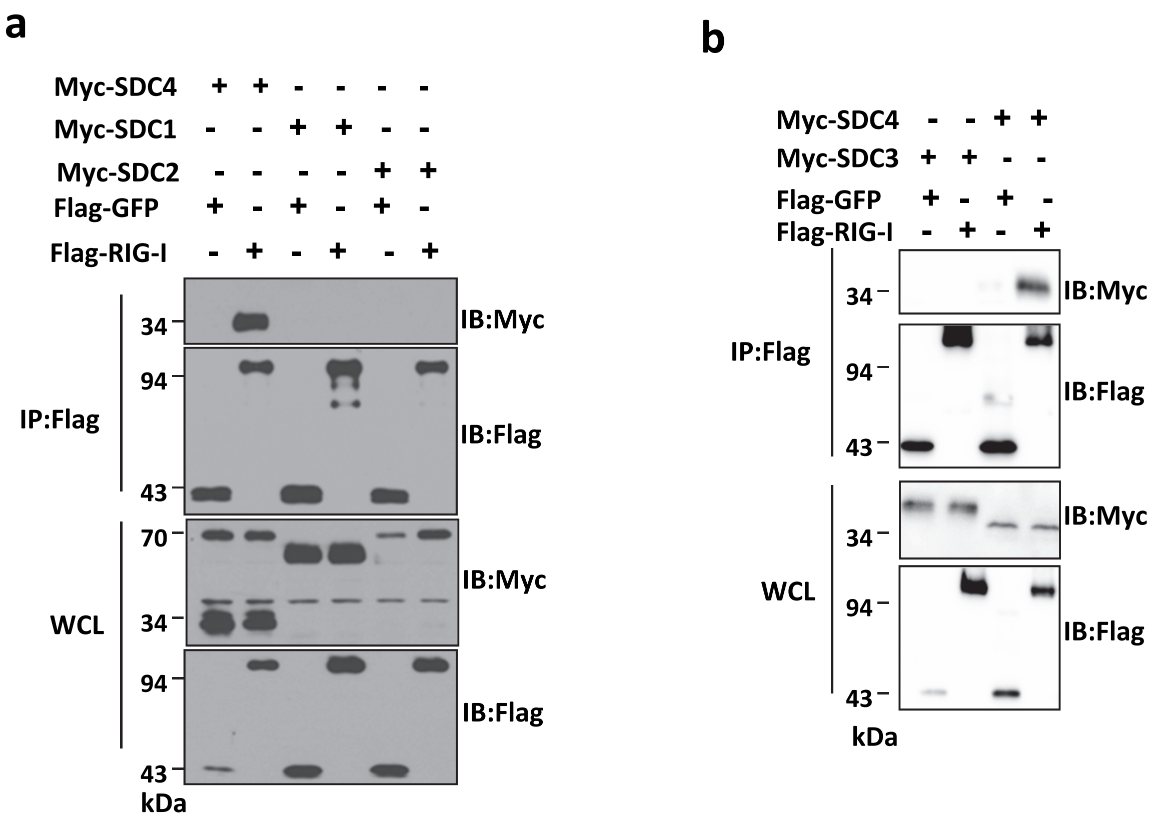

Supplementary Figure 1 RIG-I does not interact with SDC1, SDC2 and SDC3

(a-b) HEK293 cells were transfected with the indicated plasmids. Twenty-four hours post-transfection, the lysates were immunoprecipitated with anti-Flag beads, and analyzed by immunoblotting with the indicated antibodies. Bottom panels, expression of exogenous proteins in whole-cell lysates.

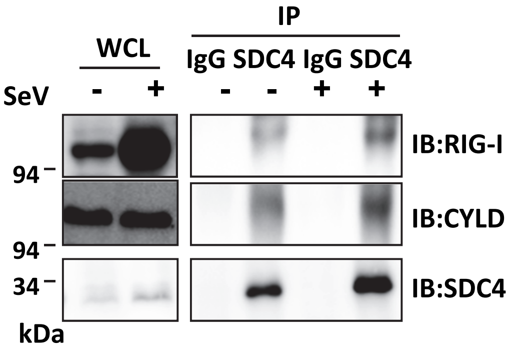

Supplementary Figure 2 SDC4 interacts with RIG-I and CYLD

HeLa cells were infected with SeV for 24 h. The lysates were incubated with IgG or anti-SDC4 antibody overnight at 4℃, immunoprecipitated with protein A/G beads and analyzed by immunoblotting with the indicated antibodies.

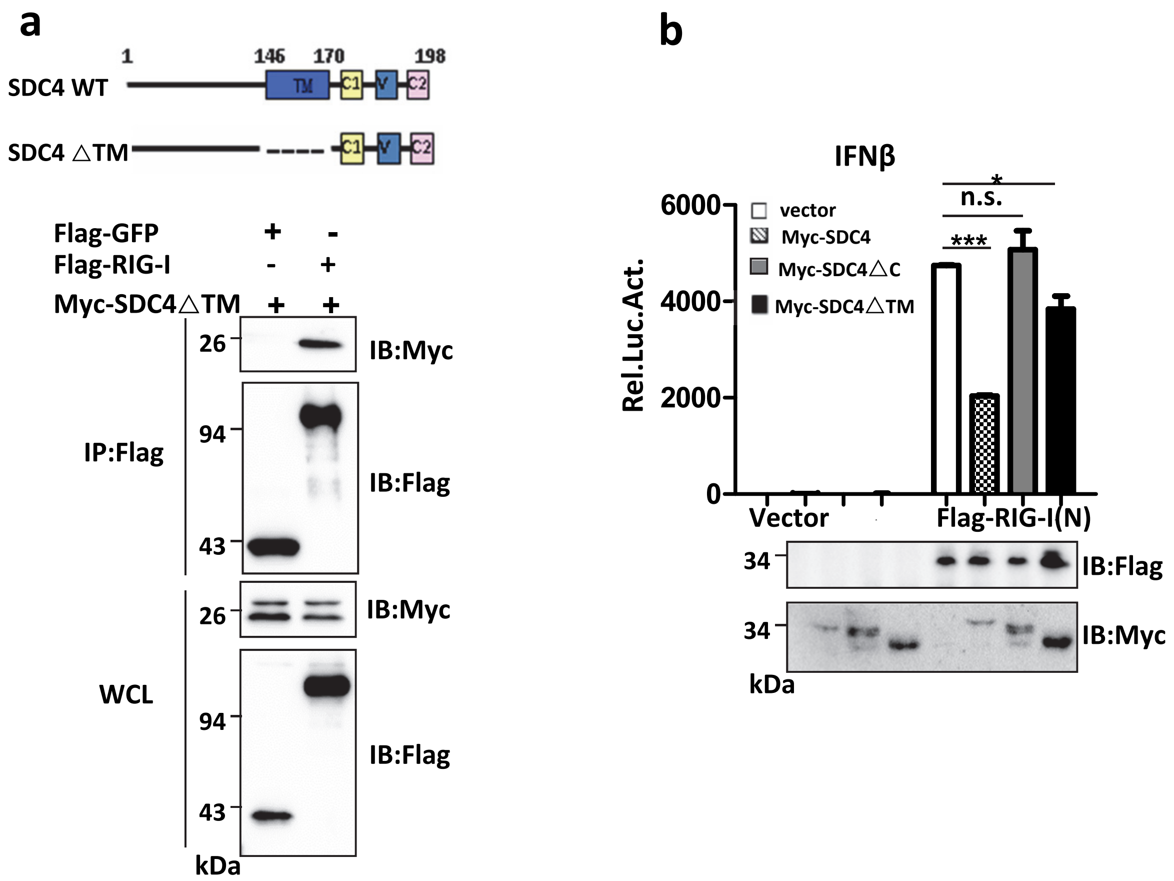

Supplementary Figure 3 The transmembrane domain is important for SDC4 to regulate the RIG-I-mediated antiviral signaling

(a) HEK293 cells were transfected with the indicated expression plasmids. Twenty-four hours post-transfection, the lysates were immunoprecipitated with anti-Flag beads, and analyzed by immunoblotting with the indicated antibodies. Bottom panel, expression of exogenous proteins in whole-cell lysates. (b) HEK293 cells were co-transfected with the indicated expression plasmids, luciferase reporter construct driven by the promoter of gene encoding IFN-β, and pRSV/LacZ as an internal control. Twenty-four hours after transfection, the cells were lysed for luciferase assays (upper panel) and immunoblotting assays (lower panels).

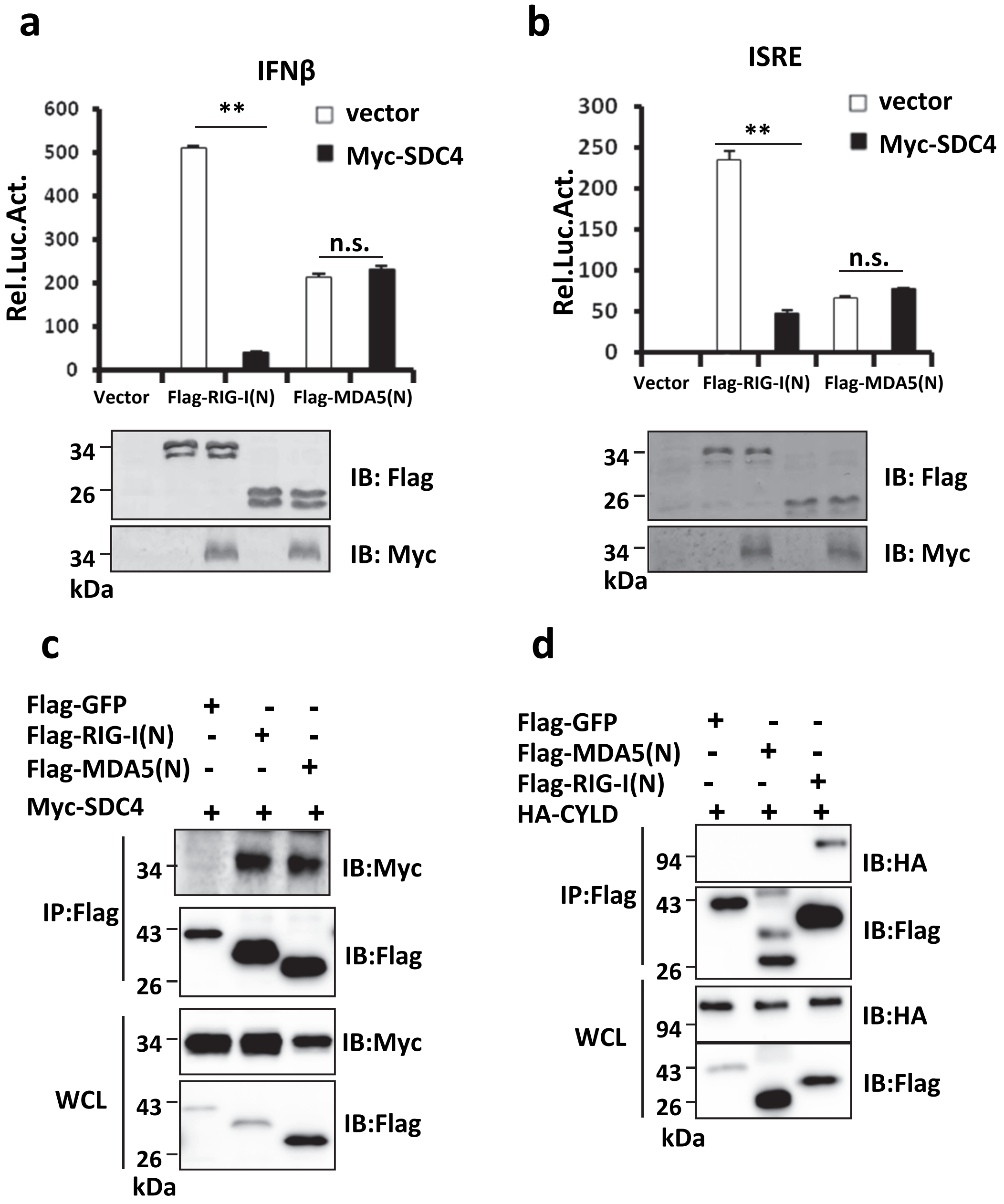

Supplementary Figure 4 SDC4 has no effect on the MDA5-mediated antiviral signaling
(a, b) HEK293 cells were co-transfected with the indicated expression plasmids and luciferase reporter constructs driven by the promoters of genes encoding IFN-β (a) or ISRE (b), and pRSV/LacZ as an internal control. Twenty-four hours after transfection, the cells were lysed for luciferase assays (upper panel) and immunoblotting assays (lower panels). (c, d) HEK293 cells were transfected with the indicated expression plasmids. Twenty-four hours post-transfection, the lysates were immunoprecipitated with anti-Flag beads, and analyzed by immunoblotting with the indicated antibodies to test the interaction between MDA5(N) with SDC4(c) and CYLD (d), respectively. Bottom panels, expression of exogenous proteins in whole-cell lysates.

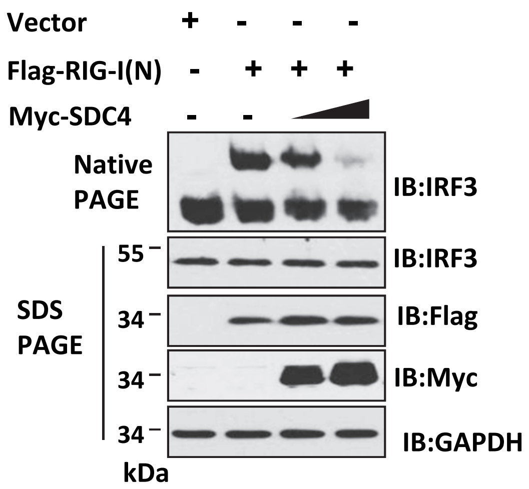

Supplementary Figure 5 Overexpression SDC4 inhibits the dimerization of IRF3 induced by RIG-I(N)

HEK293 cells were transfected with the indicated expression plasmids. Twenty-four hours after transfection, the lysates were resolved by native gel electrophoresis (upper panel) or SDS-PAGE (lower panels) and analyzed by immunoblotting with the indicated antibodies.

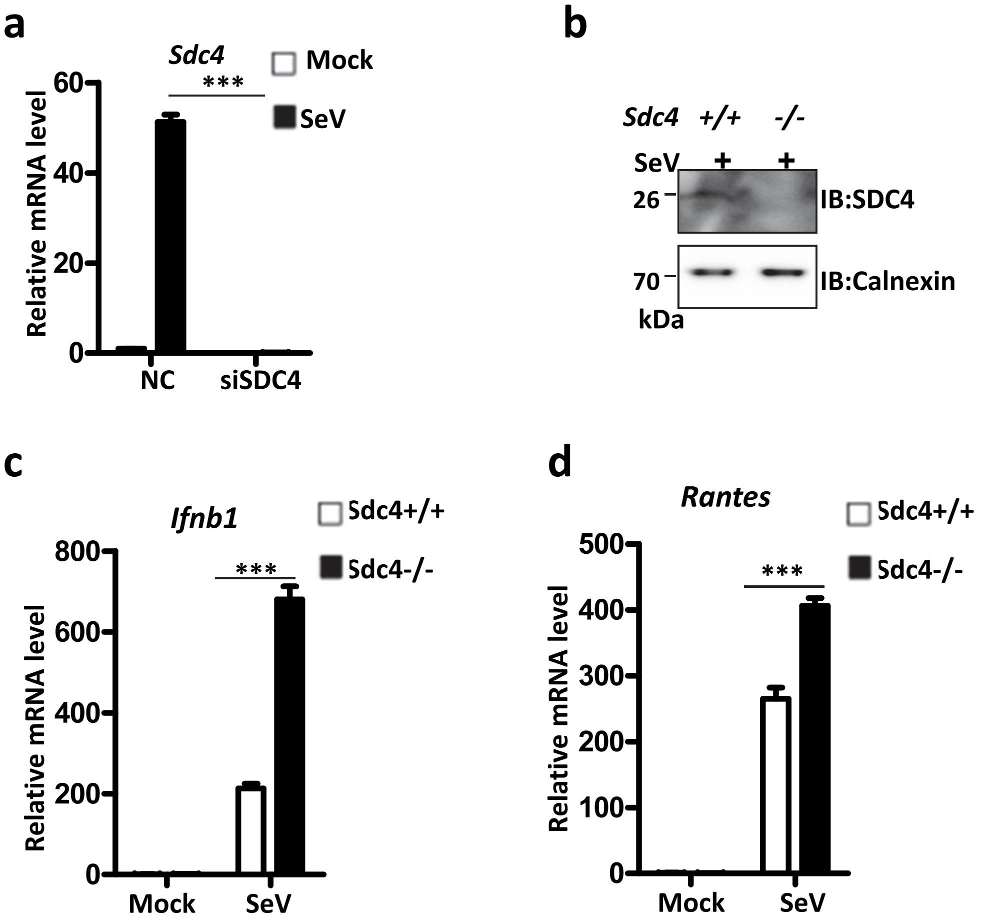

Supplementary Figure 6 Knockout of SDC4 enhances the RIG-I-mediated innate immune signaling

(a) HeLa cells were transfected with a siRNA targeting Sdc4 or NC. Thirty-six hours after transfection, the cells were infected with SeV for 9 h, and lysed to isolate RNA to measure the transcript level of Sdc4 by qRT-PCR. (b) HEK293 *Sdc4+/+* and *Sdc4-/-* cells were infected with SeV for 16 h, the cell membrane were isolated and analyzed by immunoblotting with the indicated antibodies. (c-d) HEK293 Sdc4+/+ and Sdc4-/-cells were infected with the SeV for 9 h, and the transcript levels of *Ifnb1*(c) and *Rantes* (d) were examined by qRT-PCR analysis.

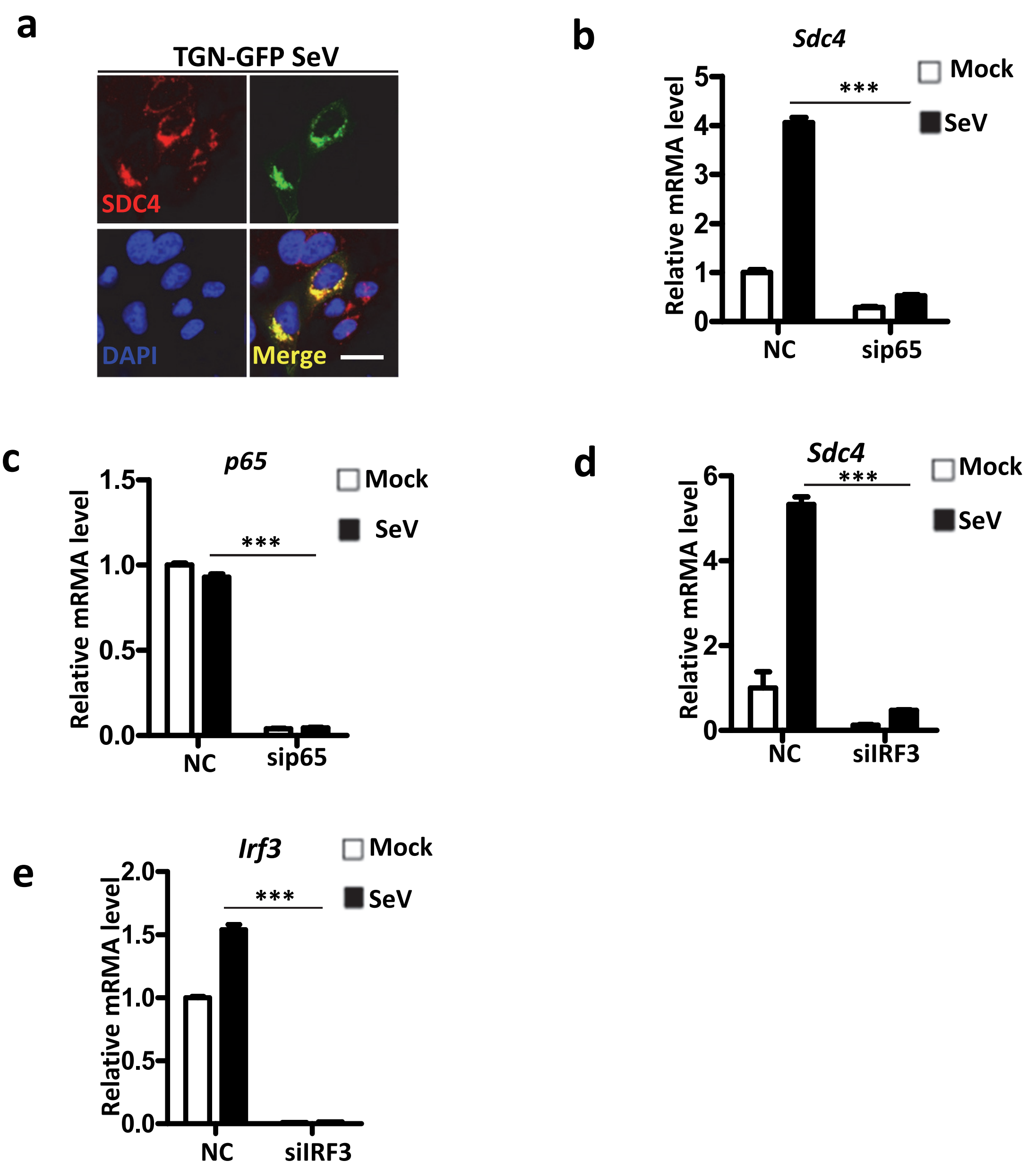

Supplementary Figure 7 p65 and IRF3 are involved in regulating SDC4 expression
(a) HeLa cells were transfected with the indicated expression plasmids. Twenty-four hours later, cells were infected or not with SeV for 16 h. The cells were fixed, stained with DAPI and anti-SDC4 antibody, and observed by confocal microscopy. Scale bar, 10μm. (b-e) HEK293 cells were transfected with a siRNA targeting p65 or IRF3. Thirty-six hours after transfection, the cells were infected with SeV for 14 h, and then lysed to measure the transcript levels of *Sdc4*(b, d), *p65*(c), and *Irf3*(e) by qRT-PCR.

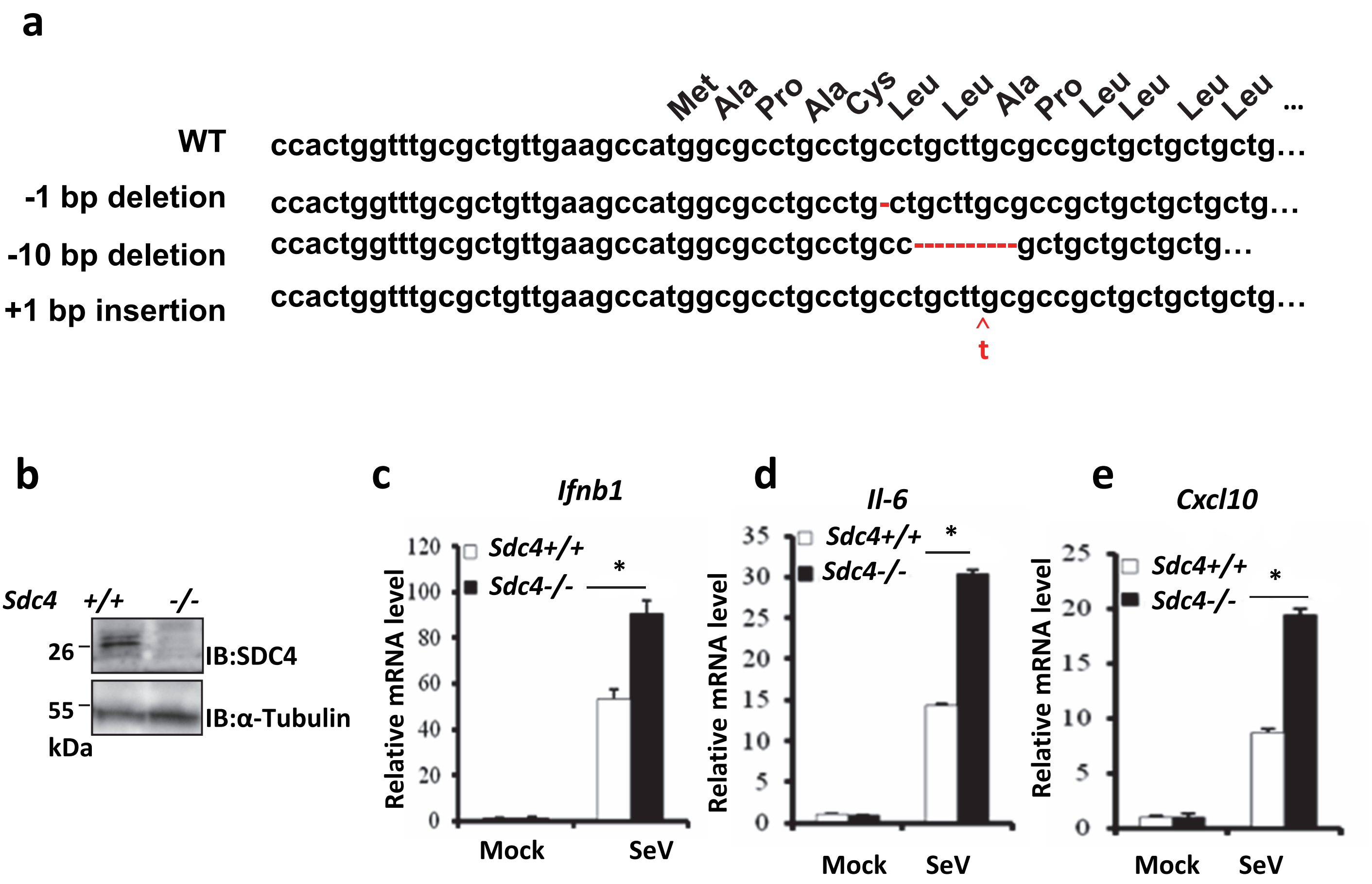

Supplementary Figure 8 Sdc4 deficiency enhances the transcript levels of antiviral genes
(a) *Sdc4* mRNA exon 1 indels caused by sgRNA targeting. (b) Protein lysates isolated from *Sdc4*+/+ and *Sdc4*–/– mouse spleen were analyzed by immunoblotting with the indicated antibodies. (c–e) Primary *Sdc4*+/+ and *Sdc4*–/– mouse bone marrow-derived macrophages were infected with the SeV for 4 h, and the transcript levels of *Ifnb1* (c), *Il6* (d), and *Cxcl10* (e) were examined by qRT-PCR analysis.

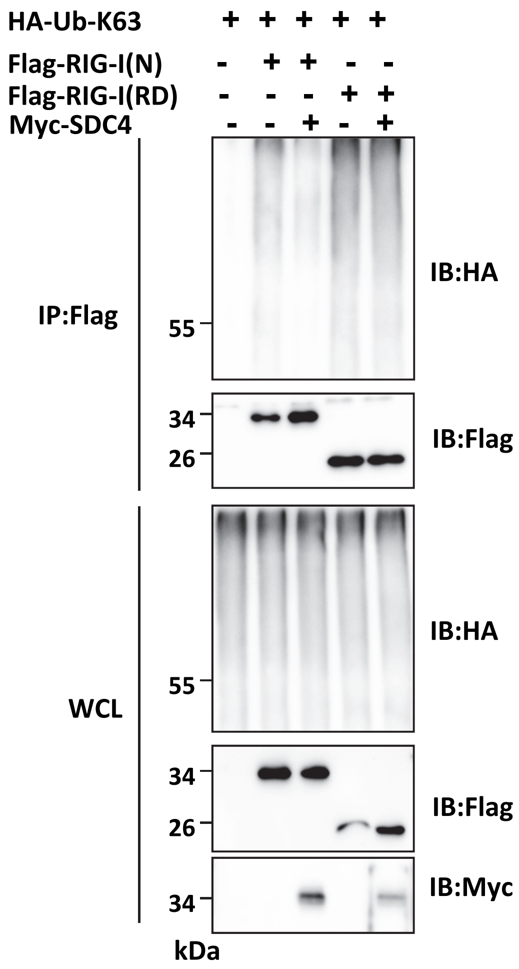

Supplementary Figure 9 SDC4 has no effect on K63-linked ubiquitination of RIG-I(RD)
HEK293 cells were transfected with the indicated expression plasmids. Twenty-four hours after transfection, cell lysates were immunoprecipitated with anti-Flag beads, and subjected to immunoblotting analysis with the indicated antibodies. The expression levels of transfected proteins in whole cell lysates were shown in the bottom panels.

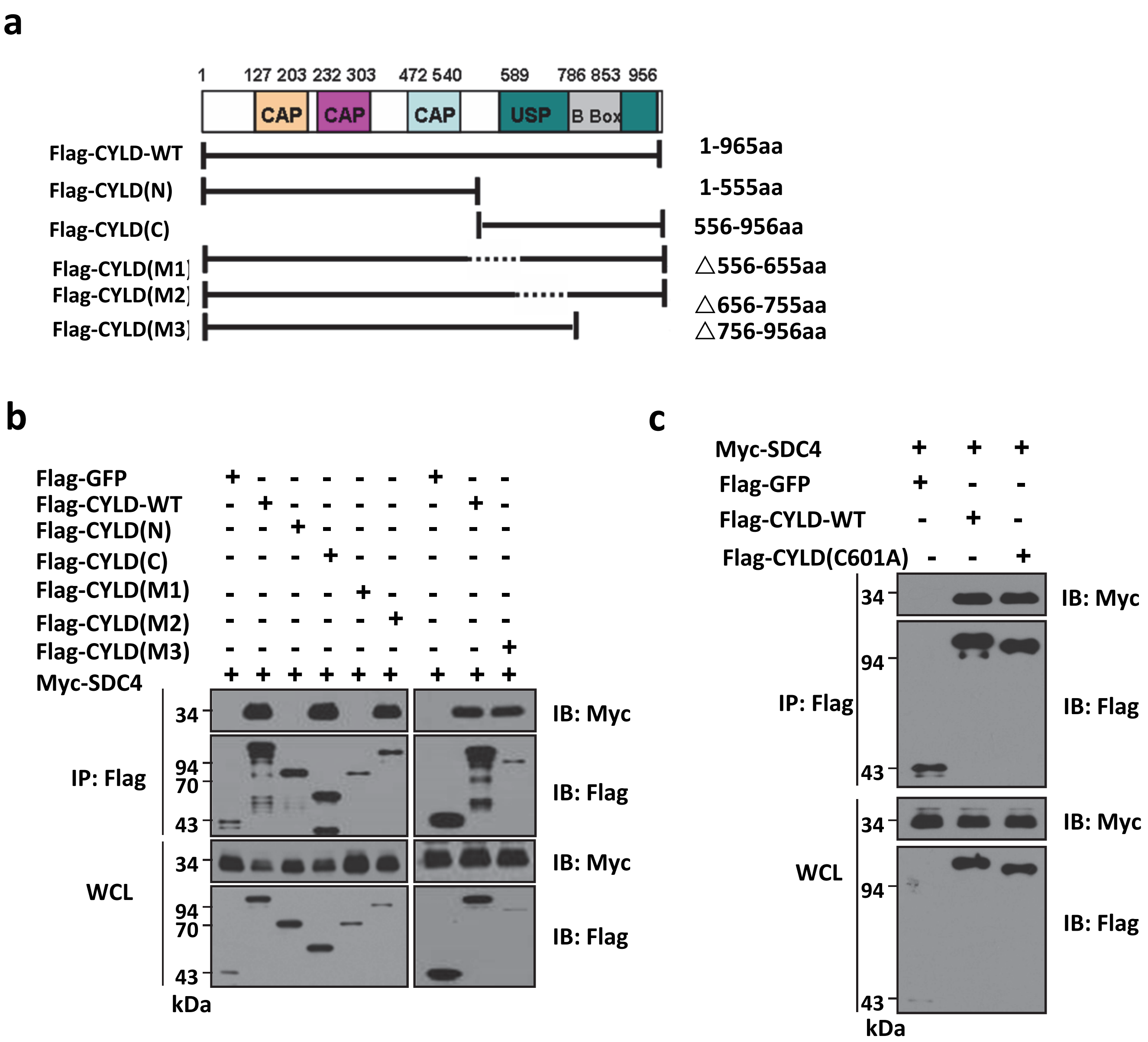

Supplementary Figure 10 The interaction between SDC4 and CYLD or its mutants

(a) Domain diagram of CYLD and its mutants. (b, c) HEK293 cells were transfected with the indicated plasmids. Twenty-four hours post-transfection, the lysates were immunoprecipitated with anti-Flag beads, and analyzed by immunoblotting with the indicated antibodies. Bottom panel, expression of exogenous proteins in whole cell lysates.

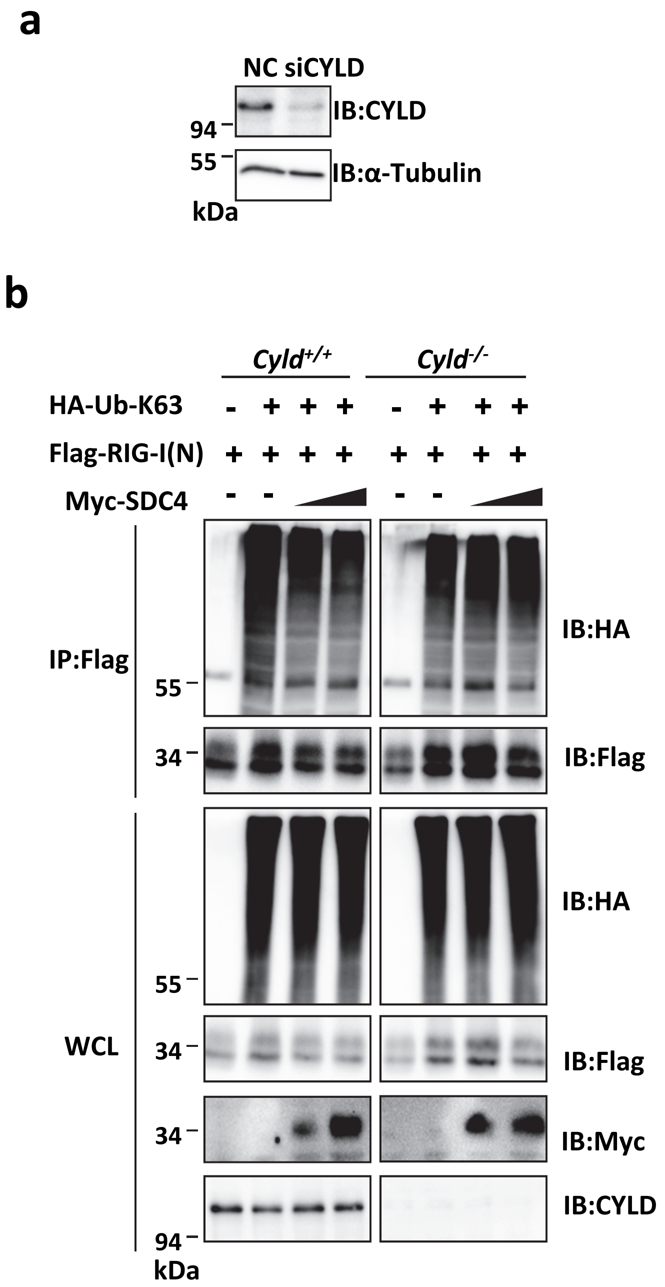

Supplementary Figure 11 SDC4 regulates the RIG-I(N) deubiquitination via CYLD
(a) HEK293 cells were transfected with a siRNA targeting CYLD or a NC. Forty-eight hours after transfection, cell lysates were analyzed by immunoblotting with the indicated antibodies. (b) HEK 293 *Cyld*$^{-/-}$ stable cells and control cells were transfected with the indicated combinations of expression plasmids. Twenty-four hours after transfection, cell lysates were immunoprecipitated with anti-Flag beads, and analyzed by immunoblotting with the indicated antibodies.

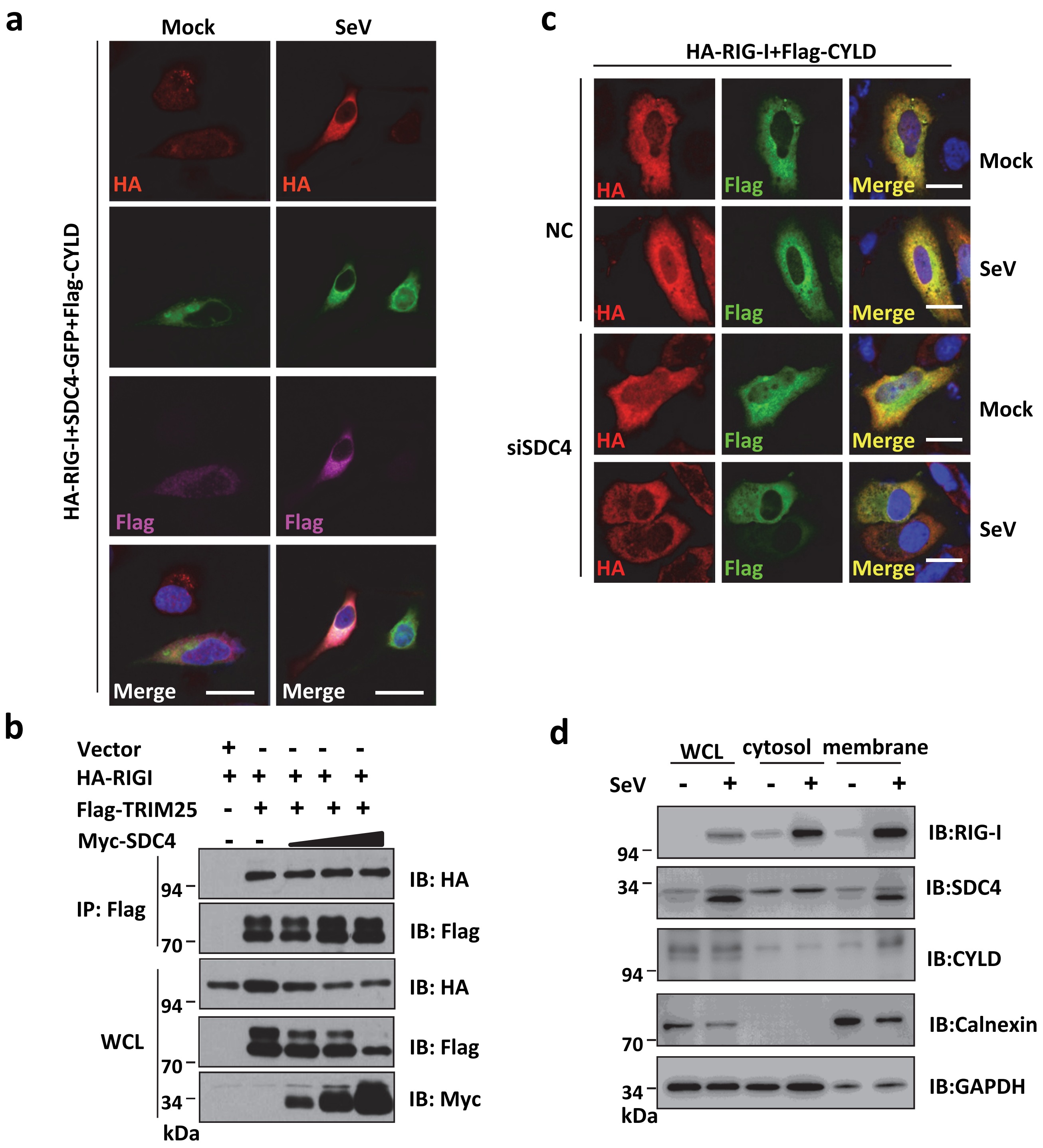

Supplementary Figure 12 Accumulation of SDC4, RIG-I and CYLD in perinuclear region after virus infection

(a) HeLa cells were transfected with the indicated plasmids. Twenty-four hours later, the cells were infected with SeV for 16 h, then fixed, stained with the indicated antibodies, and observed by confocal microscopy. Scale bar, 20µM. (b) HEK293 cells were transfected with the indicated plasmids. Twenty-four hours post-transfection, the lysates were immunoprecipitated with anti-Flag beads and analyzed by immunoblotting with the indicated antibodies. (c) HeLa cells were first transfected with siNC or siSDC4. Twenty-four hours post-transfection, the cells were transfected with the indicated expression plasmids. Twenty-four hours after the second transfection, the cells were infected with SeV for 16 h, then fixed, stained with the indicated antibodies, and observed by confocal microscopy. Scale bar, 20µM. (d) HeLa cells were infected with or without SeV for 12 h, the cell membrane were isolated. The cell cytosol and membrane component were analyzed by immunoblotting with the indicated antibodies.

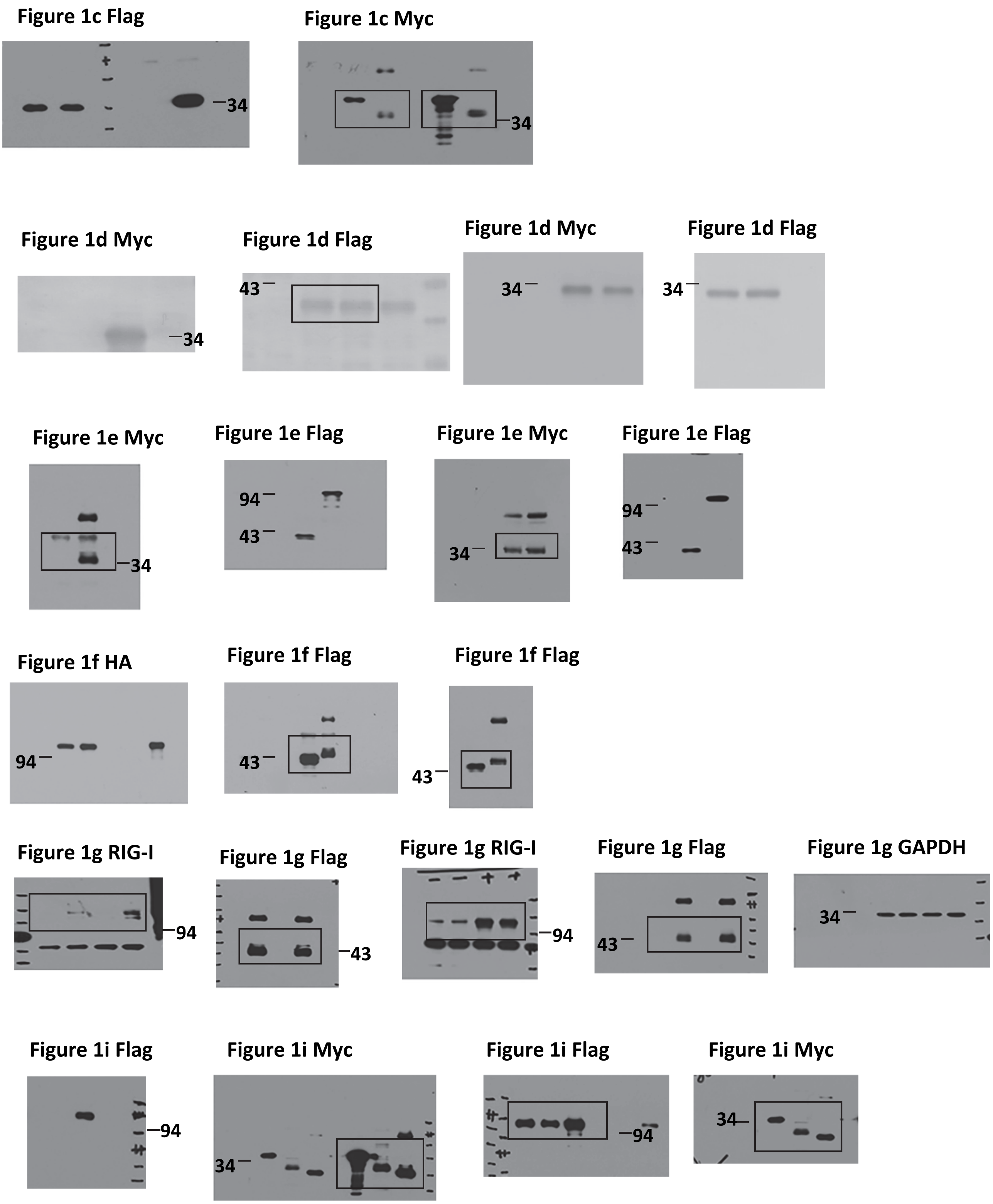

Supplementary Figure 13 Original western blots in main and supplementary figures. Panels corresponding to the figures are indicated.

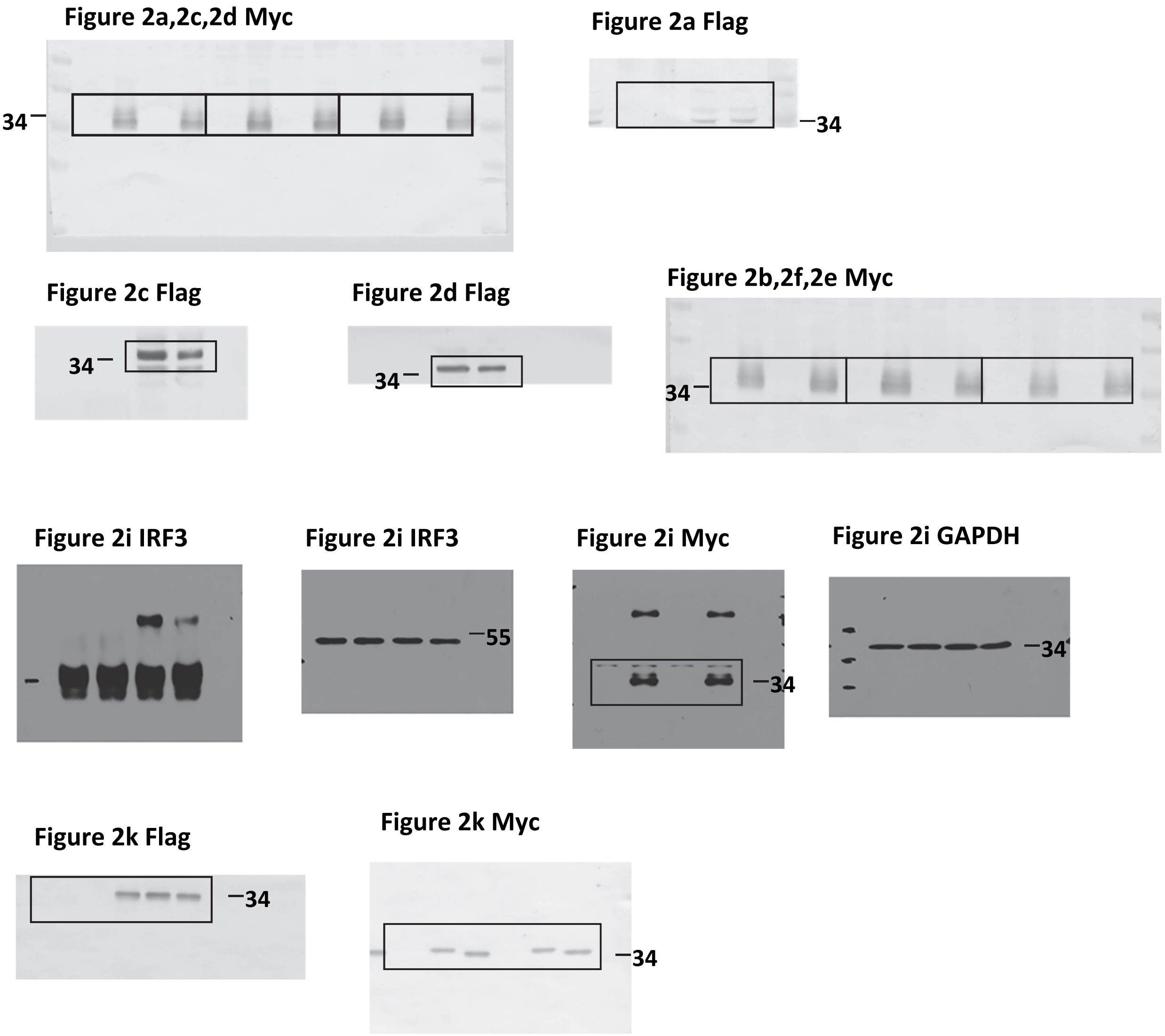

Supplementary Figure 13 continued.

Figure 3 a GAPDH SDC4

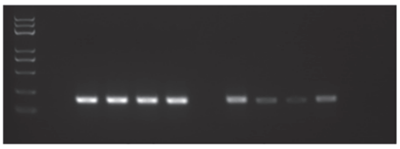

Figure 3 a SDC4

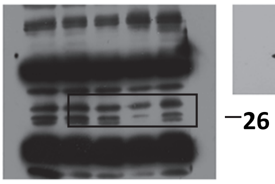

Figure 3 a α-Tubulin

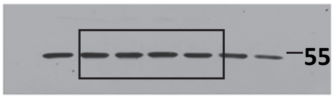

Figure 3b,3d,3c Flag

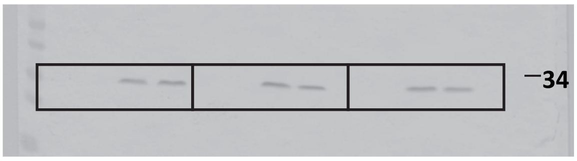

Figure 3b,3c,3d α-Tubulin

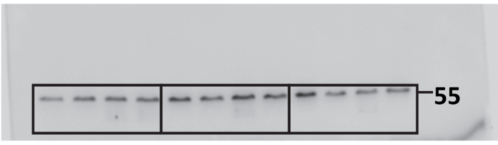

Figure 3i IRF3

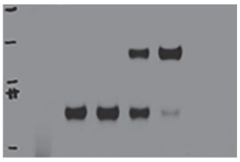

Figure 3i IRF3

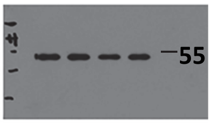

Figure 3i SDC4

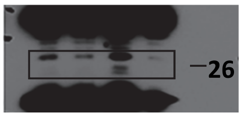

Figure 3i GAPDH

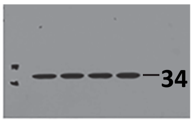

Figure 4b SDC4

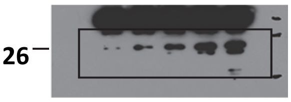

Figure 4b GAPDH

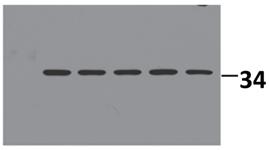

Figure 4c SDC4

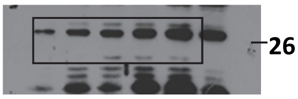

Figure 4c RIG-I

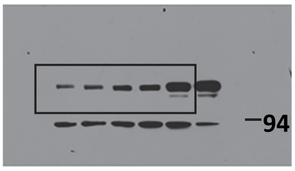

Figure 4c α-Tubulin

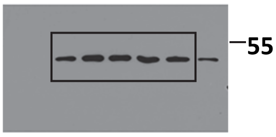

Figure 5e p-IRF3

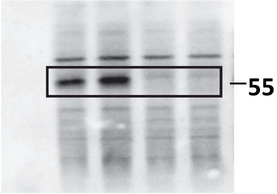

Figure 5e IRF3

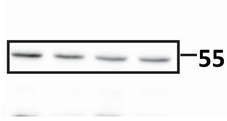

Figure 5e GAPDH

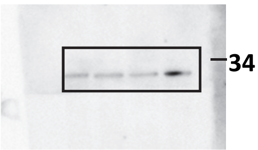

Supplementary Figure 13 continued.

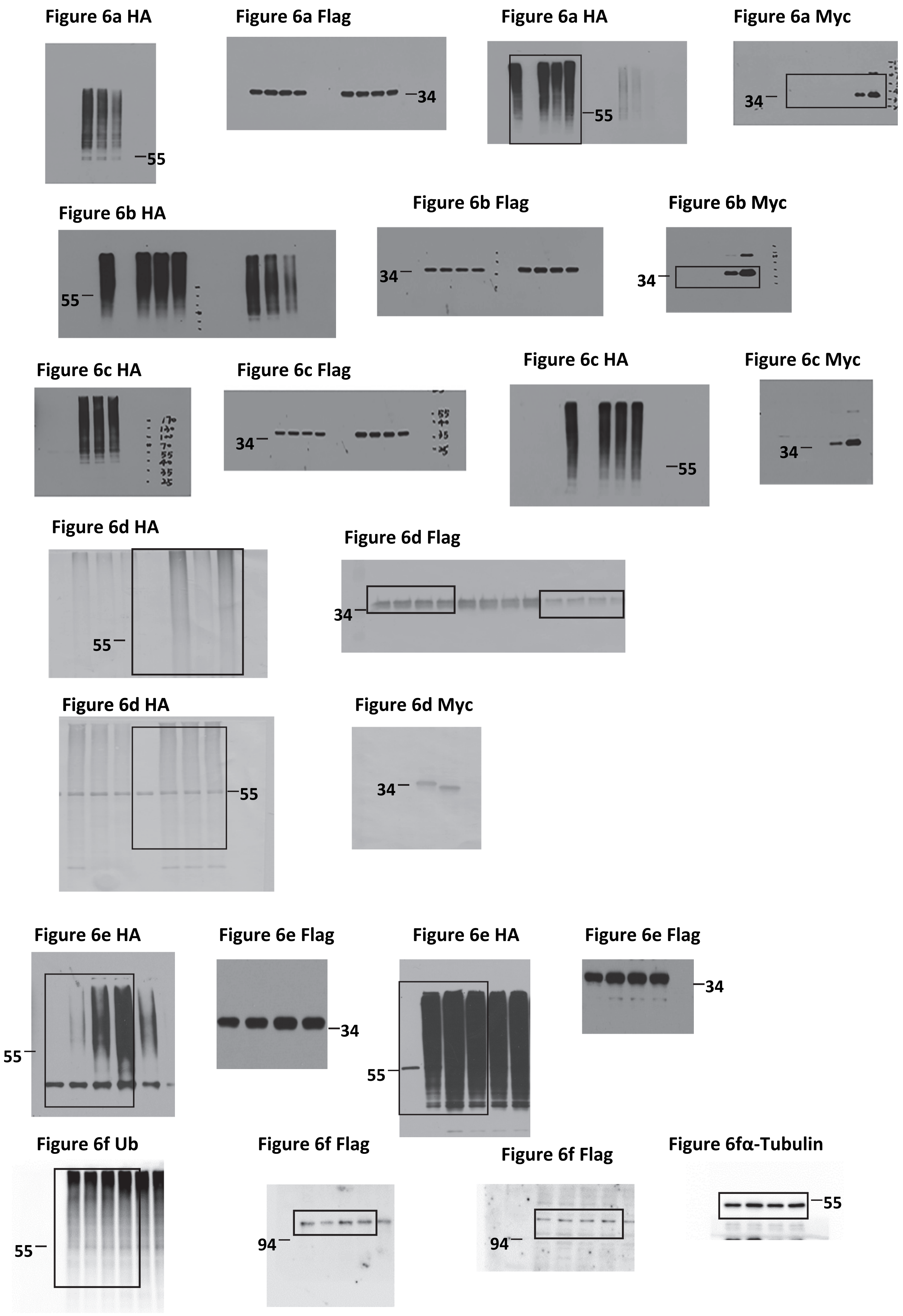

Supplementary Figure 13 continued.

Figure 7a Myc

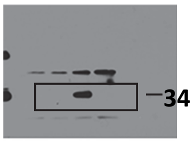

Figure 7a Flag

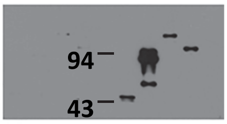

Figure 7a Myc

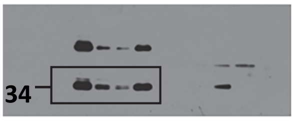

Figure 7a Flag

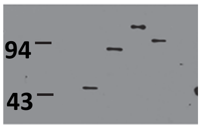

Figure 7c Myc

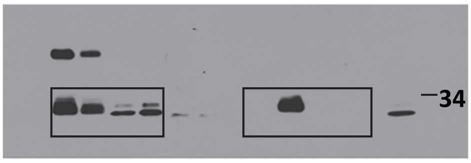

Figure 7c Flag

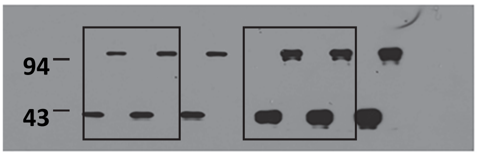

Figure 7d Myc

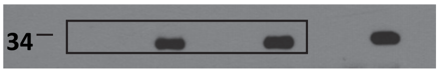

Figure 7e HA

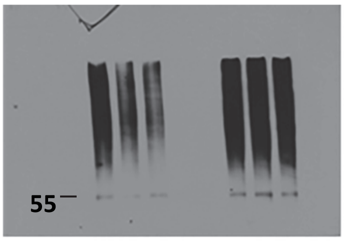

Figure 7e Flag

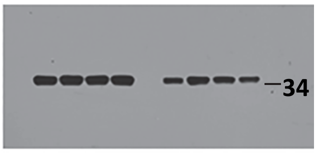

Figure 7e HA

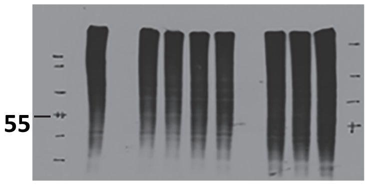

Figure 7e Flag

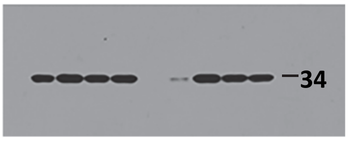

Figure 7e Myc

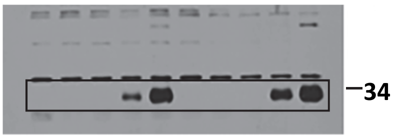

Figure 7f HA

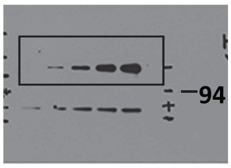

Figure 7f Flag

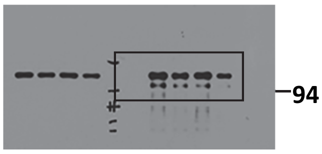

Figure 7f Myc

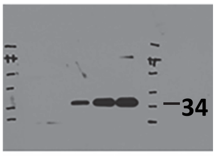

Figure 7f HA

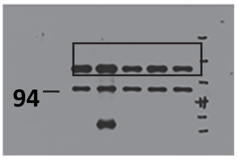

Figure 7f Flag

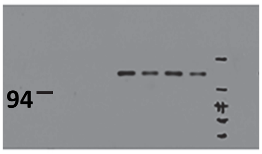

Figure 7f Myc

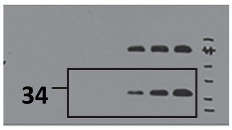

Figure 7g HA

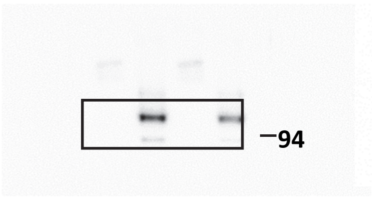

Figure 7g Flag

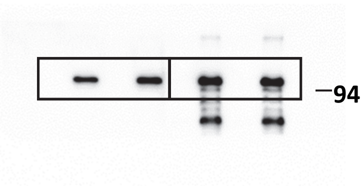

Figure 7g HA

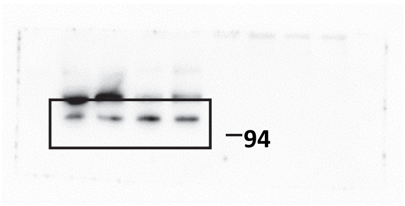

Supplementary Figure 13 continued.

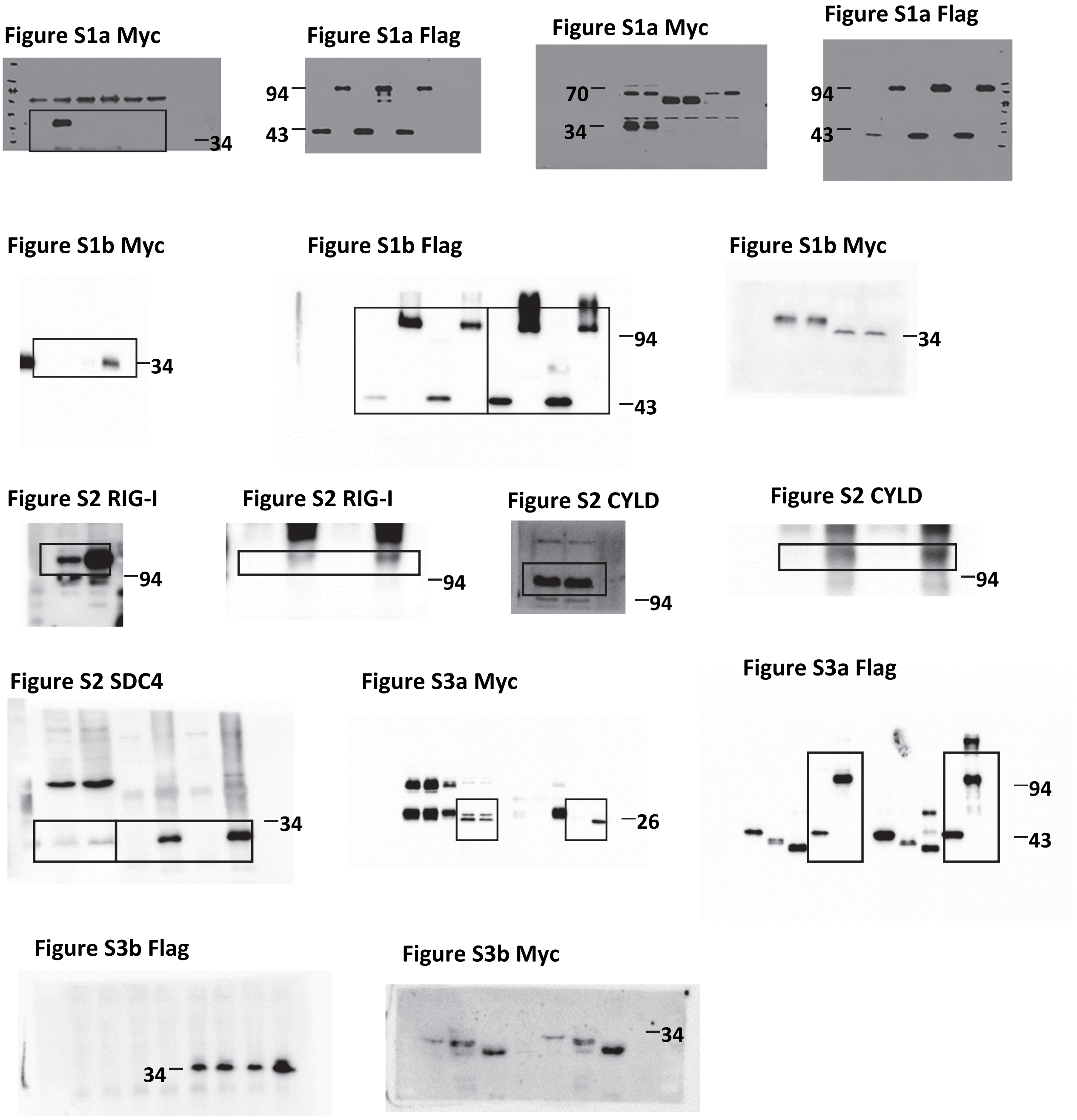

Supplementary Figure 13 continued.

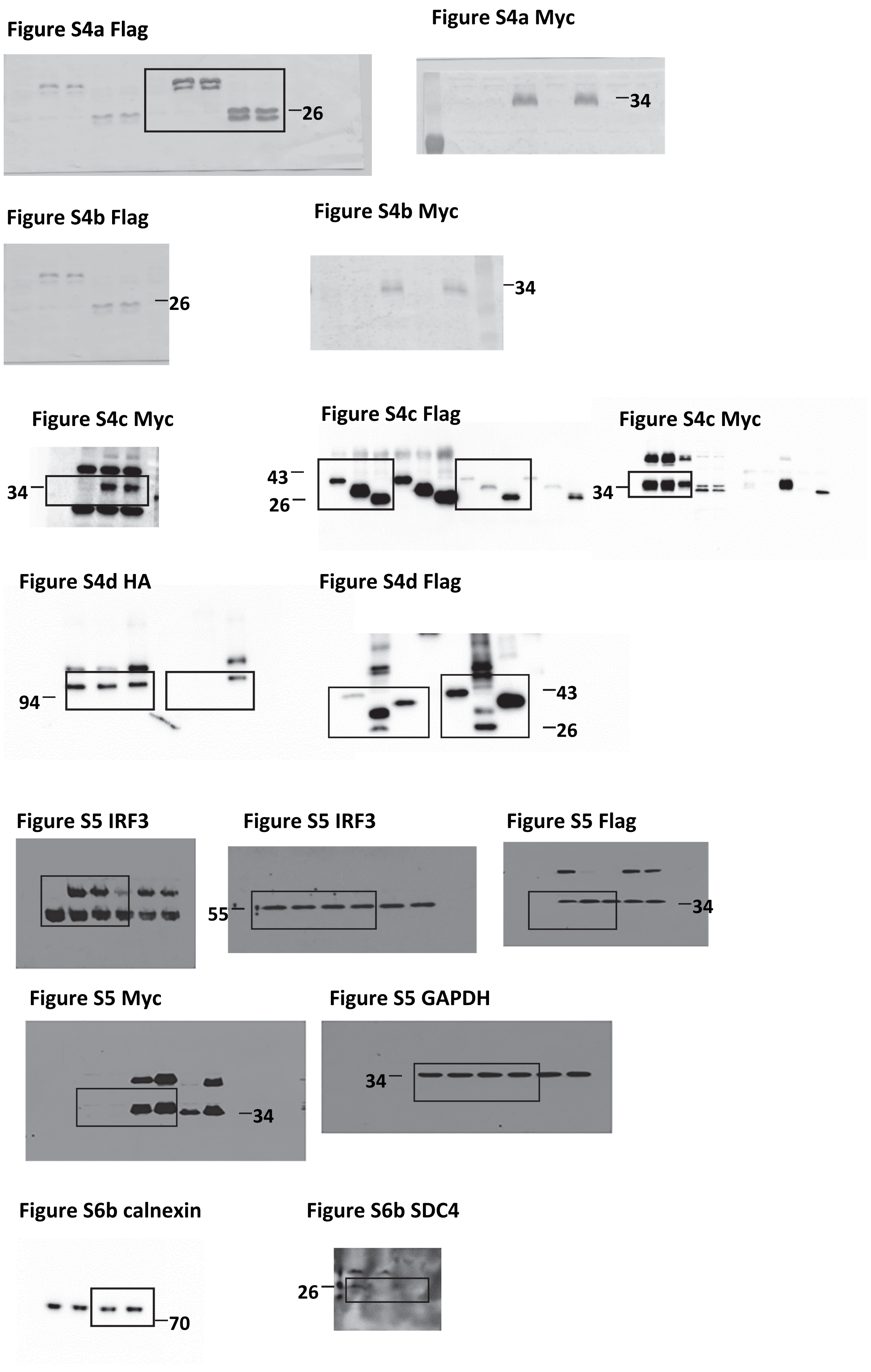

Supplementary Figure 13 continued.

Figure S8b SDC4

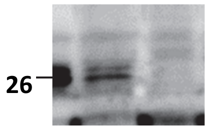

Figure S8b α-tubulin

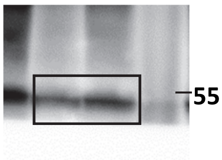

Figure S9 HA

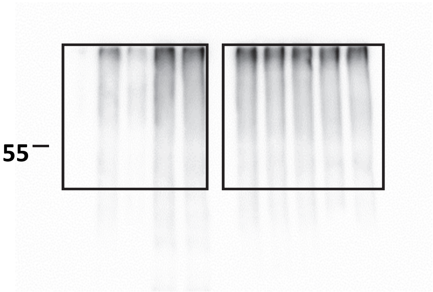

Figure S9 Flag

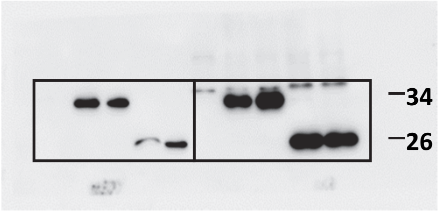

Figure S9 Myc

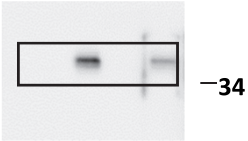

Figure S10b Myc

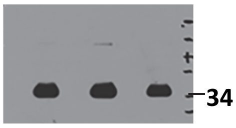

Figure S10b Myc

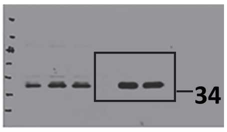

Figure S10b Flag

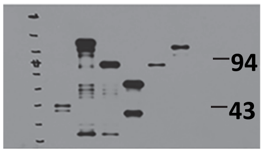

Figure S10b Flag

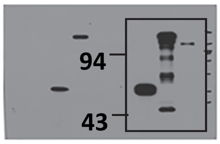

Figure S10b Myc

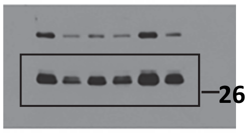

Figure S10b Myc

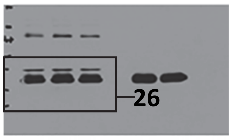

Figure S10b Flag

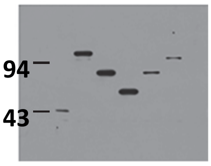

Figure S10b Flag

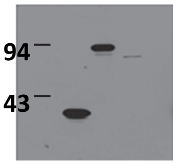

Figure S10c Myc

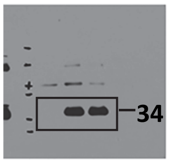

Figure S10c Flag

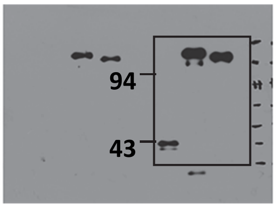

Figure S10c Myc

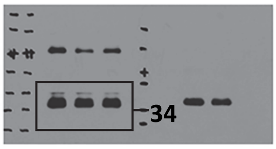

Figure S10c Flag

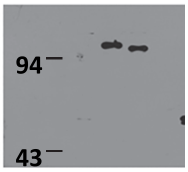

Supplementary Figure 13 continued.

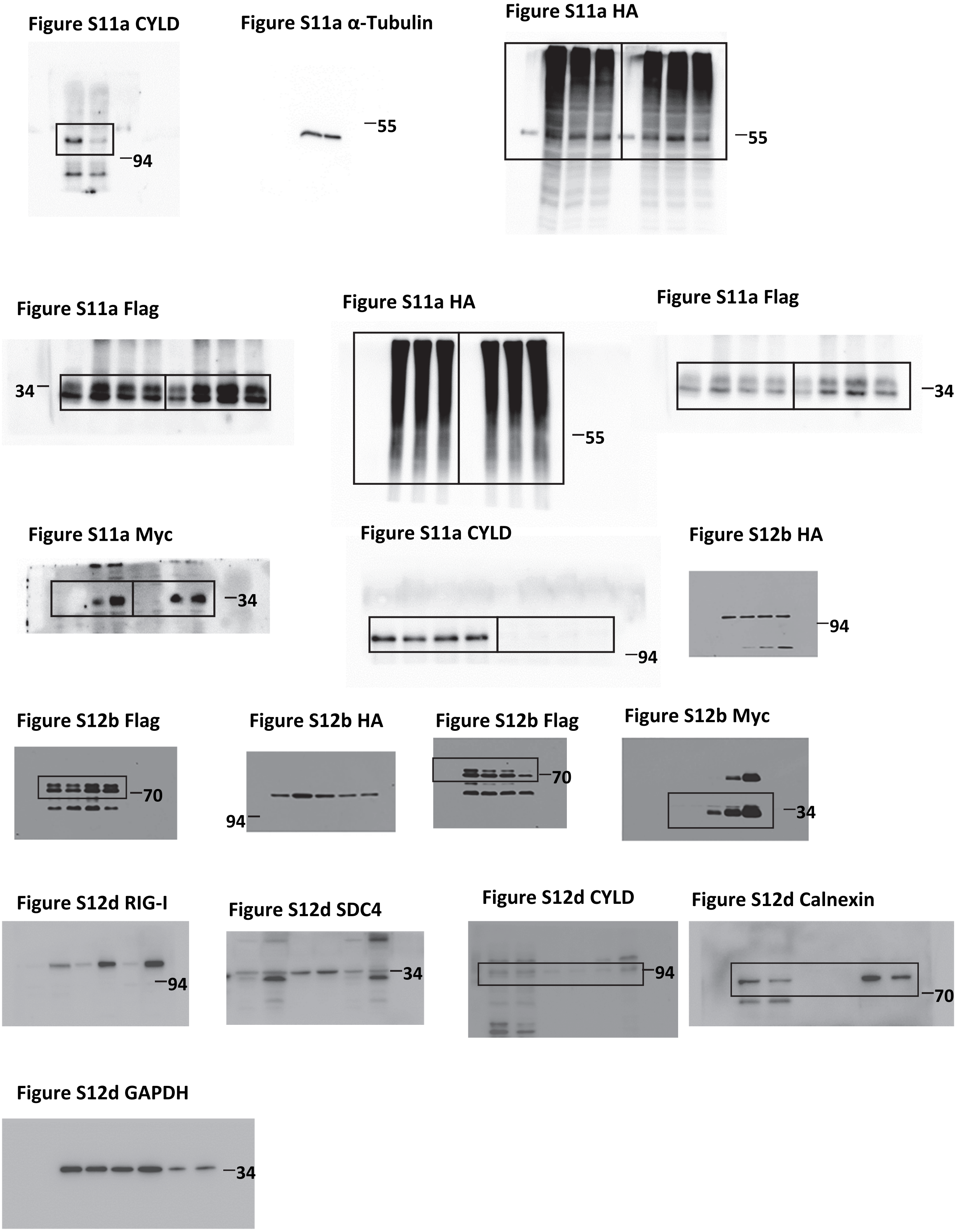

Supplementary Figure 13 continued.

Supplementary Table 1. Summary of the top 10 genome-wide potential off-target sites of sgRNA 1 and sgRNA 2

Guide	Target	Sequence	Scor e	#MM s	UCSC gene	Chr
sgRNA 1	OT1	GCAGCAGCAGCTGCGCCAGCTGG	1.2	2	NM_025276	11
	OT2	GAAGCACCAGCTGCGCAAGCGAG	0.9	3		1
	OT3	GCAACAGCAGGGGCGCAAGGCAG	0.8	3	NM_008850	11
	OT4	GCAGCAGCAGCAGCGCACGCAGG	0.6	2		11
	OT5	GCAGCAGAATCGGCACAAGCGAG	0.6	3		19
	OT6	GCAGCAGCAGAGGCGGAAGCGAG	0.6	2	NM_025816	6
	OT7	GCAGCAGCAGAGGCGGAAGCGAG	0.6	2		11
	OT8	GCGTGAGCAGCGGCGCGAGCGGG	0.6	4	NM_001177843	2
	OT9	CCGGCCGCGGCGGCGCAAGCAGG	0.5	4	NM_011441	1
	OT10	GCAGCAGCAGCTGCGGAAGCAGG	0.5	2	NM_030679	11
sgRNA 2	OT1	AGGATCTGCTTAGTCGGGGAGGG	0.6	4		9
	OT2	AGGATCGCCGTGGTCGGGGAAGG	0.5	4	NM_011715	5
	OT3	AGCAACTCTGTTGTCGGGGACAG	0.4	4		14
	OT4	GGCATCTTCTCTGTCGGGGAAGG	0.4	4		14
	OT5	AGCAGCTACTTCGTGGGGGATGG	0.4	4		7
	OT6	TACATCTTCGTCATCGGGGTGGG	0.3	4	NM_175668	7
	OT7	AGCACCTTCCTCCTCGGGGGAAG	0.2	4		8
	OT8	AGCACCTTCAGCGTTGGGGATGG	0.2	4		10
	OT9	GGCATCCTCGTCCTCGGGGCTGG	0.2	4		15
	OT10	AGCATCTACATGGTAGGGGAGAG	0.2	4		18

OT, off-target; MMs, mismatches; Chr, chromosome.

Supplementary Table 2. Sequences of locus-specific primers flanking the top 10 off-target sites of sgRNA 1 and sgRNA 2

Primer ID	Primer Sequence (5' to 3')	Primer ID	Primer Sequence (5' to 3')
sg1-OT1-s	ACTGGAGGACTGCTTATGGC	sg2-OT1-s	GCACAGCAAGCACTCTCCTA
sg1-OT1-as	TTCCCTCCTGTTCAGGACTATG	sg2-OT1-as	GCATCTGCGATGTGATTCCC
sg1-OT2-s	TGGTTTAGCACTGGGAACGA	sg2-OT2-s	AGGGCTTAGGACACCAGAGT
sg1-OT2-as	ACAGGGAAAGGCAAAGCGA	sg2-OT2-as	AATGGAGCTGGTGCTGAGTG
sg1-OT3-s	GGGAAAGTTGGAGAAGAGGC A	sg2-OT3-s	GCACGAATTCAGACCCTGTTC
sg1-OT3-as	AGCAGCCAGTCTGAAGAAGC	sg2-OT3-as	TGCCACGTAAGGTATGGACC
sg1-OT4-s	TGCAGTGTTAGGCATTGTGGT	sg2-OT4-s	AACAAATGCCTTCTGACAGTGG
sg1-OT4-as	TGGAGAGAGGTTTCCCTTCCT	sg2-OT4-as	AAGAAGCTTTGGAAGAGGCCA
sg1-OT5-s	TTCGCCGGATTTCTCAGGTT	sg2-OT5-s	GGTGCTACACTACCTTGGCA
sg1-OT5-as	CAGGAGCGCTTTCCTGGTAG	sg2-OT5-as	ATGACTTCCCCAGAGGGACT
sg1-OT6-s	GCTATGACAGTTCTTGGGGCT	sg2-OT6-s	CCTTTGCTTAGCCTGCCACA
sg1-OT6-as	AAGAGAGCTTCGTCACCACG	sg2-OT6-as	CACAGGAAGGCGATGCTGATA
sg1-OT7-s	TCTCCCTGTGAAAGGTTTGGT	sg2-OT7-s	GCTCTTGGGCTGCTCATACT
sg1-OT7-as	TGGCGGTAAACTTCTGCTCT	sg2-OT7-as	AGAGGGTAAGAGTAAGTCCCCT
sg1-OT8-s	GTGACTTGGGGTGTCCTTGG	sg2-OT8-s	CAGTCATGCCTGCTTAACCTT
sg1-OT8-as	AAACCAGCTTCCCATTCGTCC	sg2-OT8-as	GCGTGGTCACTAAACACTCAC
sg1-OT9-s	CCCATGTGCGGAGACATCA	sg2-OT9-s	AGGCAGAGCCCCTTGTAATAG
sg1-OT9-as	CCCCTTCTAGGCGTCCTGTA	sg2-OT9-as	TGAGCCTGAGAGAAGCAACC
sg1-OT10-s	ATGTTCCCTAAGGCGACAGAC	sg2-OT10-s	CTCAGCCCACTAGTCCCTTTG
sg1-OT10-as	ATATCCCTGCCCTGCCCTTTA	sg2-OT10-as	CAGACACAGAAGGAAACCCCAA
